# Air-Breakdown Triboelectric Nanogenerator Inspired by Transistor Architecture for Low-Force Human–Machine Interfaces

**DOI:** 10.1007/s40820-026-02103-0

**Published:** 2026-02-11

**Authors:** Karthikeyan Munirathinam, Longlong Li, Arunkumar Shanmugasundaram, Jongsung Park, Dong-Weon Lee

**Affiliations:** 1 MEMS and Nanotechnology Laboratory, School of Mechanical System Engineering, Chonnam National University, Gwangju, 61186 Republic of Korea; 2 Advanced Medical Device Research Center for Cardiovascular Disease, Chonnam National University, Gwangju, 61186 Republic of Korea; 3 Center for Next-Generation Research and Development, Chonnam National University, Gwangju, 61186 Republic of Korea; 4 Department of Precision Mechanical Engineering, Kyungpook National University, Sangju, 37224 Republic of Korea

**Keywords:** Skin electrons, Air breakdown, Electrostatic discharge, Low contact force, Human–machine interfaces

## Abstract

Human–machine interface (HMI) systems require energy harvesters that can operate efficiently under low contact forces, yet conventional tactile triboelectric nanogenerators (TENGs) suffer from low surface charge density and unstable output. Here, we propose a human skin electric field-induced air-breakdown TENG (AB-TENG) with a transistor-inspired architecture. The device employs a base terminal to collect electrons from human skin via an ionized air channel formed by air breakdown, enabling efficient conversion of the skin’s electric field through two operational modes: indirect (accumulated output) and direct (instant high output). In direct mode, the AB-TENG delivers 165 V at 2 N and 290 V at 24 N, with a peak power of 22 mW—22 times higher than conventional tactile TENGs. Practical utility is demonstrated through a self-powered infrared remote control and an ultrathin keyboard. This work establishes a new design paradigm that transforms air breakdown from a limitation into a functional mechanism, advancing skin-electricity-enhanced thin-film TENGs toward next-generation self-sustaining HMI platforms.

**Supplementary Information:**

The online version contains supplementary material available at 10.1007/s40820-026-02103-0.

## Highlights

- An air-breakdown triboelectric nanogenerator (AB-TENG) is proposed with a transistor-inspired architecture to achieve high electrical output from the electrostatic discharge of skin electrons at a low contact force.
- The working contact force of AB-TENG is compatible with the day-to-day human–machine interface systems, enabling the fabrication of next-generation thin electronics.
- Demonstration of AB-TENG-based self-powered infrared remote control and an ultrathin self-powered keyboard with a thickness of 600 µm.

**Supplementary Information:**

The online version contains supplementary material available at 10.1007/s40820-026-02103-0.

## Graphical abstract

**Figure:**
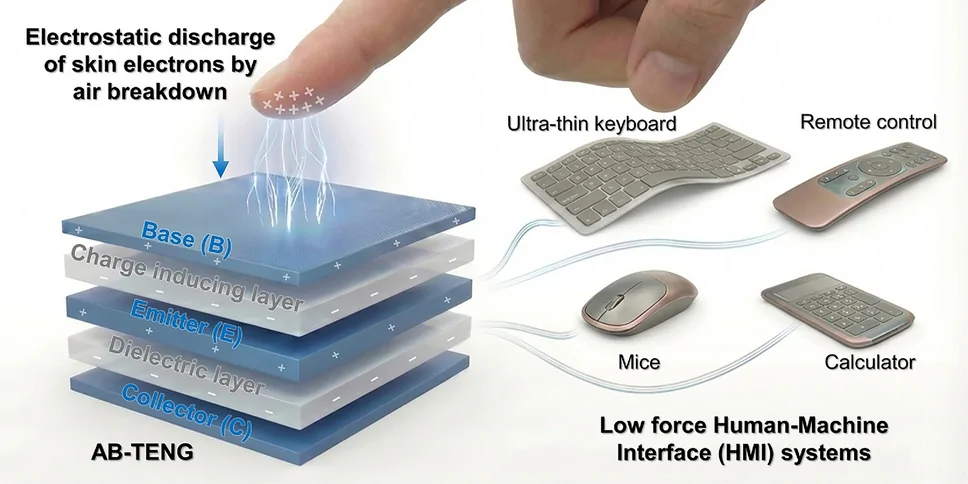

## Introduction

Advances in micro/nanotechnology are driving human–machine interface (HMI) systems toward miniaturized, intelligent, autonomous platforms for next-generation electronics [1–7]. The integration of artificial intelligence and 5G technologies has further expanded the capabilities of HMI systems by enhancing responsiveness, compactness, and user accessibility [8–11]. However, most current HMI devices remain passive and dependent on external power sources, which restricts their potential for truly self-sustained operation [12, 13]. Although human skin holds the highest positive charge in the triboelectric series, the lack of innovative conversion technologies leads to substantial charge loss during the HMI process [14, 15]. Furthermore, the contact force exerted during the daily use of electronic devices such as keyboards, mice, and remote controls is extremely low, making it difficult to convert this weak mechanical force into usable electricity efficiently. Therefore, next-generation HMI systems require active energy-harvesting technologies that can effectively operate under such low contact forces.

Triboelectric nanogenerators (TENGs) are emerging as a promising approach for harvesting electricity from human activities such as walking, finger tapping, or even sleeping motions [16–24]. TENGs operate on the coupling of contact electrification and electrostatic induction [25–27]. When two different materials repeatedly come into contact and separate, charge transfer occurs due to differences in electron affinities, with human skin being a highly effective triboelectric material. By utilising the skin’s natural triboelectric effect, the human body accumulates positive charges through contact triboelectrification with surrounding materials, having free electrons to lose, especially at the skin surface [28–31]. The buildup of charges on the skin can even produce a static shock by quickly releasing electrons to a conductive object in contact [32–35]. Several TENGs have been developed to harvest skin static energy via electrostatic induction [36–40]. Most skin-based TENGs typically use the skin as one of the triboelectric layers, with polymers such as PDMS, PTFE, PET, and Kapton serving as other materials [41–44]. Consequently, the high static charges accumulated on the skin through triboelectrification with these polymers can cause electrostatic discharge (ESD) of electrons to the environment due to air breakdown, which reduces energy conversion efficiency in skin-contact TENGs. Researchers are exploring ways to exploit this phenomenon, stabilising surface charges and leveraging ESD rather than being hindered by air breakdown.

Although air breakdown may occur during contact electrification in TENGs, it has typically been regarded as a disruptive factor and thus overlooked in most studies [45, 46]. This phenomenon usually results in sudden and sporadic electrical outputs, making stability and control difficult.

More recently, however, researchers have attempted to transform this drawback into an opportunity. For instance, Luo et al. proposed a direct current triboelectric nanogenerator (DC-TENG) that harvests contact–separation energy via ionized air channels formed by air breakdown [47], while Liu et al. introduced a TENG designed to produce a constant current using electrostatic breakdown [48]. The performance of the reported air-breakdown TENGs depends on the charge density of the dielectric materials used. Moreover, such approaches often require relatively high contact force and still yield unstable outputs, which significantly limit their applicability in practical HMI scenarios [49, 50]. While reported methods use air breakdown during the triboelectrification of two materials as a mean to achieve a high output current, the air breakdown during the ESD of human skin electrons has yet to be investigated. Given that typical human–machine interactions involve only 2–15 N of force, it is crucial to develop energy-harvesting devices capable of reliably utilizing the ESD of skin electrons to achieve an active HMI system under low-force conditions [51–56].

To address these challenges, this study proposes a human skin electric field-induced air-breakdown triboelectric nanogenerator (AB-TENG). The device employs a transistor-inspired structure, featuring a base terminal exposed to ambient air, which collects electrostatically discharged electrons from the human skin through an air-breakdown effect triggered by finger motion. Operating in two distinct modes, indirect and direct, the AB-TENG generates electricity through different charge transfer mechanisms. In the indirect mode, the electric field induced by the finger modulates charge distribution between the emitter and collector via electrostatic induction, resulting in a potential difference and alternating current output. In the direct mode, electrons collected by the base terminal are directly transferred to the emitter through a base–emitter contact point and subsequently delivered to the collector. This dual-mode operation ensures efficient charge transfer and high output even at low contact force, overcoming the intrinsic limitations of conventional TENGs. To demonstrate the practicality of our approach, we further developed two representative applications: a self-powered infrared (IR) remote control for wireless LED operation and a 600 µm-thick ultrathin keyboard. The air-breakdown strategy implemented in AB-TENG thus opens new avenues for effectively utilizing the human skin’s electric field and advancing triboelectric energy-harvesting technologies for next-generation HMI platforms.

## Experimental Section

### Fabrication of Air-Breakdown Triboelectric Nanogenerator

The air-breakdown triboelectric nanogenerator (AB-TENG) is designed based on a transistor-inspired architecture. It consists of five layers, namely base, emitter, charge-inducing layer, dielectric layer, and collector. The base, emitter, and collector terminals are made of a 2.5 cm × 2.5 cm copper electrode sheet, while the dielectric layer (charge-inducing layer) is made with a slightly larger dimension than the electrodes (3 cm × 3 cm). The final AB-TENG device is assembled by sequentially fixing the individual layers, including the collector, dielectric layer, emitter, charge-inducing layer, and base, from bottom to top. To enable direct-mode operation, a 4 mm × 4 mm hole was made in the charge-inducing layer, allowing the base and emitter terminals to be electrically connected. In the application section, to create an IR remote control, four AB-TENGs, a microcontroller, and an IR transmitter LED are mounted on a printed circuit board (PCB) measuring 8 cm × 5 cm. An ultrathin self-powered keyboard is fabricated on an A4 sheet featuring 4 rows and 8 columns, with a total of 30-character keys (AB-TENGs). All the devices are wired on the backside of the A4 paper.

### Characterization and Measurement

The electrical characterization of the AB-TENG was achieved by the contact and separation of a human finger. The electrical parameters, i.e., the voltage and current generated by the AB-TENG, were measured using a Tektronix TDS 2014B oscilloscope. The low-noise current amplifier (Stanford Research Systems, SR570) was used for the current measurements. The contact force of the human finger was measured using a commercial force sensor. The energy harvested by the AB-TENG was used to power LEDs. Furthermore, next-generation thin electronics were developed using a commercial ATtiny85 microcontroller, IR transmitter and receiver LEDs, an Arduino Uno board, and the Arduino programming platform.

## Results and Discussion

### Structure, Working Mechanism, and Evaluation of AB-TENG

**Fig. 1:**
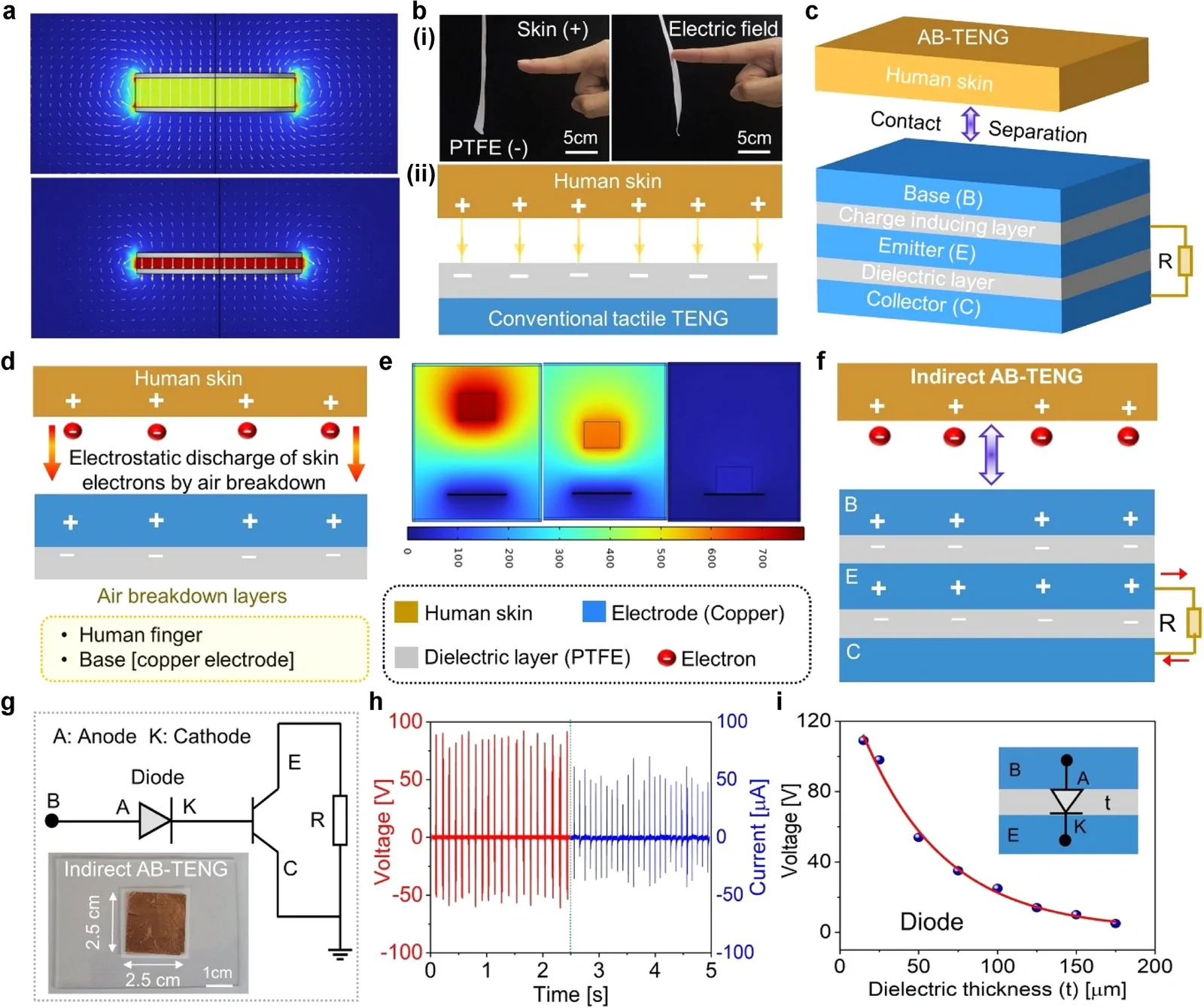
Air-breakdown triboelectric nanogenerator (AB-TENG).** a** Illustration of electric field formation between the oppositely charged human skin and PTFE a) using COMSOL simulation and **b(i)** using real-time demonstration. **b(ii)** Conventional tactile TENG used to convert the human skin electric field into electricity. **c** Schematic illustration of the proposed AB-TENG structure. **d** Air-breakdown mechanism between human skin and the base terminal. **e** COMSOL simulation of potential distribution during the motion of a human finger toward the AB-TENG. **f, g** Schematic and its electronic circuit equivalent of the proposed AB-TENG in indirect mode. (Inset in **g** shows the photograph of the indirect AB-TENG.) **h** Voltage and current output. **i** Influence of dielectric thickness on the output voltage of AB-TENG in indirect mode

The existing triboelectric touchpad can make seamless contact with human stimuli and produce reliable electrical output from the human skin electric field. However, the interaction mechanisms of most touchpads are passive, primarily because of their low-power output density. Considering the fact that the contact force of the human finger with HMI systems is in a few newtons, the use of the human skin electric field plays a key role in achieving active interactions with next-generation electronics. According to the triboelectricity of the human body, the motion of the body generates electrostatic charges due to triboelectrification with surrounding materials, such as shoes, resistive floors, clothing, chair textiles, walking across a carpet, and general movement in a dry environment [57–59]. During triboelectrification, the body accumulates positive charges from the neighboring materials and readily gives up more free electrons. Therefore, the skin has a higher positive position in the triboelectric series. The generated electrostatic charges can be converted into electricity through the classical electrostatic induction (ESI) and electrostatic discharge (ESD) processes. In the ESI process, human skin uses the positive charges to establish a strong electric field with dielectric materials. The formation of the electric field between human skin and the negatively charged dielectric material is tested by simulation (Fig. 1a). It shows that when the human skin is kept at a distance of more than 20 mm away from the dielectric material, the electric field interaction between them becomes negligible, as the field strength significantly weakens in air. However, when the skin is moved toward the dielectric surface, the charges are polarized, and a strong electric field is formed between them (Fig. S1)**.** Further**,** a physical method is conducted to test the presence of an electric field in the human finger **(**Fig. 1bi). The positively charged human finger is gently moved toward a negatively charged PTFE (dielectric) tape with a thickness of 75 µm. As the finger approaches the PTFE tape, an electric field is formed between them due to the force of attraction (Movie S1). While the conventional TENGs utilize the human skin electric field to polarize the charges in the dielectric layer, they convert them into electricity by placing an electrode below the dielectric surface using the ESI process (Fig. 1bii). This electric field accelerates the free electrons in the skin to collide with air molecules and ionize them. While this process leads to an electrical breakdown, transforming the air from an insulator to a conductor, the skin electrons are discharged to the environment due to the air-breakdown effect. Therefore, the energy conversion efficiency of the conventional TENGs is low. Further, the interaction mechanism between skin and the dielectric layers is asymmetrical, causing abrupt and intermittent output decline due to the negative effect of air breakdown.

On the other hand, in the ESD process, the electrons in human skin are rapidly transferred to a conductive material due to the potential gradient between them [29]. If a human finger and the conductor are close to each other, the charges are transferred through the arc discharge due to the air-breakdown effect, while the charges from the human skin are directly transferred to the conductor through the contact discharge process upon physical contact. This discharge of electrons from the human body relates to the principles of physics regarding electrostatic discharge or corona discharge. By taking advantage of ESD, an air-breakdown triboelectric nanogenerator (AB-TENG) is proposed to overcome the limitations of conventional TENG and achieve high output power density from a low contact force (Fig. 1c). The AB-TENG consists of five layers, namely the base (*B*) fixed at the top of the device, the charge-inducing layer, the emitter (*E*), the dielectric layer, and the collector (*C*). The structure of AB-TENG is designed based on a transistor architecture; however, the base terminal plays a key role in converting the electric field from a human finger into electricity via air breakdown. The air breakdown occurs between the human finger and the base terminal, which is exposed to the atmosphere (Fig. 1d). Here, the base terminal needs a field effect to modulate AB-TENG operation. As the main component, the human finger acts as a source of electric field, where the AB-TENG utilizes the air-breakdown-induced ionized air channel to discharge electrons from the finger to the base terminal. For a significant electric field to form that can ionize air (dielectric breakdown), the human body should first accumulate an electrostatic charge. This typically happens through the triboelectrification of human skin with surrounding materials. During triboelectrification, the human skin accumulates positive charges from the surrounding materials and thereby loses electrons to them. As described in the literature, the human skin is similar to a typical TENG structure, with the outermost layer of skin (epidermis) acting as a dielectric layer with a high impedance of up to 10 MΩ, whereas the low resistance (300 Ω) tissues beneath the skin behave like an electrode [32]. Here, the positive charges generated by the human skin are stored and conducted by the human body to the surface of the finger. Therefore, the finger surface holds more free electrons to lose to the base terminal of AB-TENG. As the base terminal is connected to ground through the emitter and collector, there is a potential gradient between the finger (high potential) and the base terminal (low/ground potential) due to the difference in the electron affinities between them. Hence, the free electrons from the finger surface are transferred to the base terminal through the ESD process, equalizing the equilibrium between them. Initially, there is no physical contact between the finger surface and the base terminal. As the human finger approaches the AB-TENG, it establishes an electric field between the finger and the base terminal. The strength of the electric field is verified by the simulation results (Fig. S2a). It shows that as the distance between the human finger and the base terminal decreases, the strength of the electric field increases. Once the human finger and base terminal are close enough to each other, the air breakdown occurs owing to the influence of the electric field. Such a breakdown allows the electrons to flow partially from the finger to the base terminal of AB-TENG through the ionized air channel between them. According to Paschen’s law, the gas-breakdown voltage depends on air pressure and distance [60]. So, when the human finger moves closer, the intense electric field ionizes more air molecules and transfers more electrons from the human finger to the base terminal. However, the gas-breakdown voltage deviates from the classical form of Paschen’s law when the microscale gap is in the range of 1–10 μm [49]. Therefore, when the distance between the human finger and the base terminal is less than 10 μm, the air breakdown is dominated by electron field emission due to high electric fields at microscale distances [61]. Depending on the strength of the electric field (distance), the electrons discharged from the human finger to AB-TENG vary linearly. On the other hand, when the finger physically contacts the base terminal in a subsequent motion, electrons are fully transferred from the human finger through the contact discharge process, increasing the surface charge density of AB-TENG. The ESD process during the motion of the human finger toward the base terminal is verified using the simulation results of potential distribution (Figs. 1e and S2b). Though the physical contact of the finger fully discharges the electrons to the base terminal, the human skin retains a steady supply of free electrons due to the triboelectrification of the human body with surrounding materials. Therefore, the human skin always has a potential difference with the base electrode and transfers electrons continuously to maintain the sustainable operation of AB-TENG during the subsequent contact–separation process. The electrons transferred from the human finger to the base terminal through ESD are collected by AB-TENG in two different modes, namely indirect and direct.

The device schematic diagram and the initial charge condition of AB-TENG in indirect mode are shown in Fig. 1f. The triboelectrification of the human body with surrounding materials accumulates positive charge and holds free electrons to lose on the finger surface. The charge-induction layer acts as a stable reservoir of electrostatic charge, inducing charge redistribution in the underlying emitter–collector structure. Although the finger does not triboelectrically contact the charge-inducing layer, the PTFE surface maintains a fixed negative charge relative to the top metal electrode. This persistent charge produces a strong electrostatic field that modulates the potential of the emitter and collector. The electric charge in the emitter and the collector terminals polarizes the polymer dielectric layer between them. During operation, the motion of the human finger ionizes the air channel and transfers electrons to the base terminal through ESD. The electrons transferred from the finger enhance the surface charge in the charge-inducing layer, playing a key role in creating potential differences between the emitter and the collector. A detailed schematic of AB-TENG in indirect mode, illustrating potential distribution and electron flow during the alternating motion of the human finger, is shown in Fig. S3. As the human finger approaches the base terminal, a strong electric field is formed between them. At this stage, the electrons that tune the operation of the AB-TENG are transferred from the human finger to the base terminal through the ionized air channel. This process enhances the strength of the electric field in the charge-inducing layer and induces electrons to flow from the emitter surface to the collector through an induction process. When the finger moves further and intensifies the electric field in the charge-inducing layer, it induces more electrons to flow from the emitter to the collector owing to a higher potential difference between them. Once the finger moves forward and contacts the base terminal, AB-TENG produces a maximum output. Subsequently, when the human finger moves away from the base terminal, the electric field strength in the charge-inducing layer reduces, and the electrons flow back from the collector to the emitter. As the finger moves further away and disrupts the electric field interaction with the base terminal, the induction process and potential difference die out between emitter and collector, allowing no electrons to flow from the collector. However, the skin readily has free electrons for the subsequent contact–separation process due to triboelectrification with the surrounding materials. According to the above mechanism of AB-TENG in indirect mode, the ESD of electrons from the human finger to the base terminal increases the electric field strength in the charge-inducing layer and controls the charge flow between the emitter and collector through the induction process.

As the electron flow between emitter and collector depends on the electrostatic induction process, the AB-TENG in indirect mode behaves like a composite circuit with a diode connected in series to a transistor (Fig. 1g). The inset image (Fig. 1g) shows the photograph of the AB-TENG operating in indirect mode. The structure of AB-TENG is designed in a square shape with dimensions of 2.5 cm × 2.5 cm. The charge-inducing layer and the dielectric layer were made larger than the terminals to avoid electrical contact between the electrodes. Figure 1h shows the output voltage and current produced by the motion of a human finger with a maximum contact force of 24 N. The AB-TENG in indirect mode produces a maximum voltage of 95 V and a current of 70 µA. The influence of the charge-inducing layer on the induction process of AB-TENG is confirmed by selecting different negative (PTFE and Kapton) and positive (polyamide and paper) triboelectric materials (Fig. S4). Despite having high charge density due to a strong electric field, the charge-inducing layer serves as a surface barrier to electron flow owing to the indirect induction mechanism. Hence, the output voltage decreases with an increase in the thickness of the charge-inducing layer (Figs. 1i and S5). Therefore, the output power of AB-TENG in indirect mode suffers from greater energy loss and exhibits reduced energy conversion efficiency.

### Evaluation of AB-TENG in Direct Mode

**Fig. 2:**
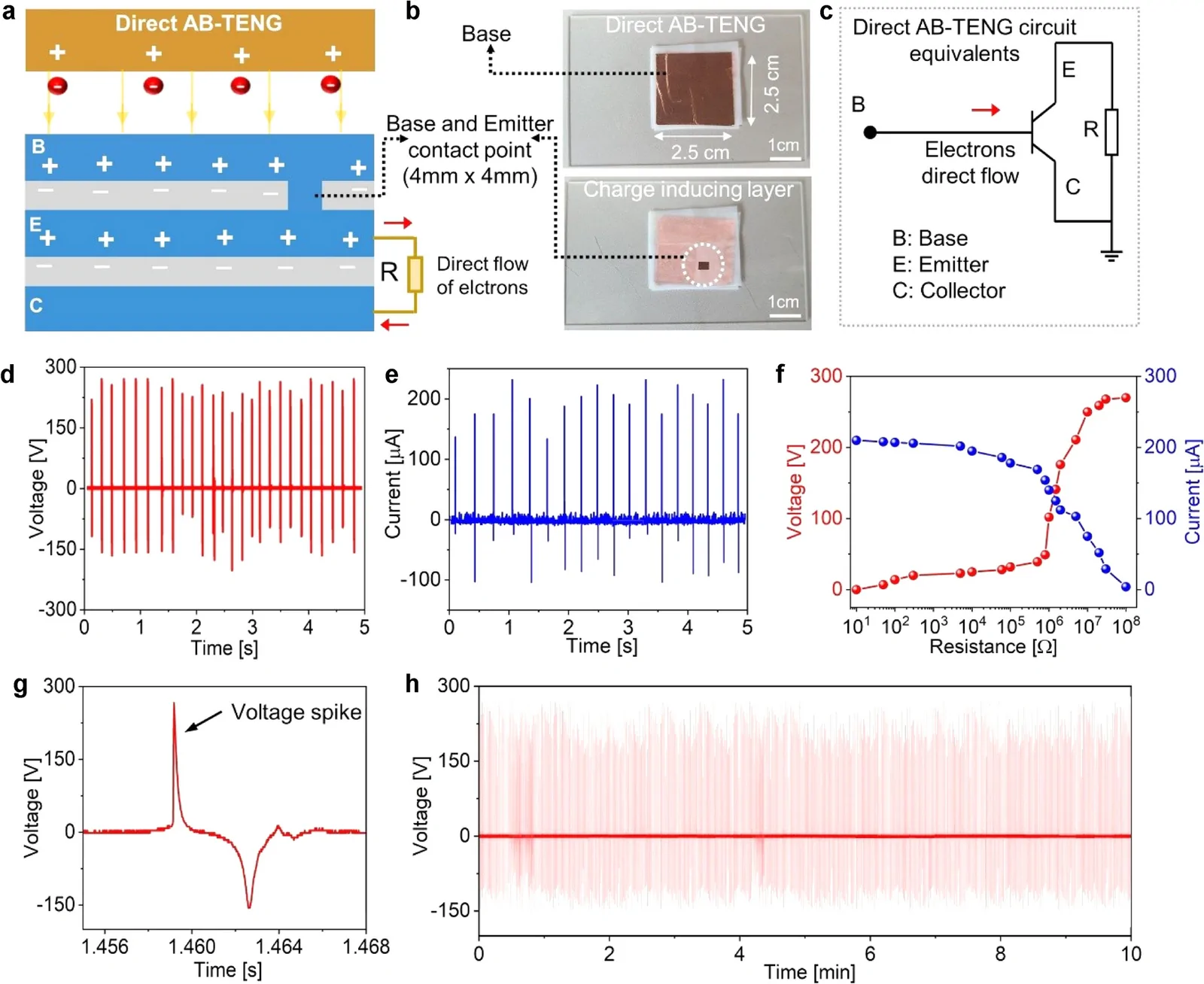
Air-breakdown triboelectric nanogenerator (AB-TENG) in direct mode.** a** Schematic illustration of the AB-TENG structure. **b** Photograph of the AB-TENG along with the charge-inducing layer displaying the base–emitter contact point. **c** Electronic circuit equivalents of AB-TENG in direct mode. **d, e** Output voltage and current waveforms. **f** Load resistance versus the electrical output. **g** Output voltage spike of the AB-TENG. **h** Durability and long-term stability test on AB-TENG by continuously tapping the base terminal for 2000 cycles

To overcome the limitations of the indirect mode, the AB-TENG is reported in direct mode (Fig. 2a). The overall structure of the direct AB-TENG and the initial charge condition are similar to those of the indirect AB-TENG; however, the base and emitter terminals are electrically connected to achieve the direct flow of electrons between the base and collector. Figure 2b shows a photograph of AB-TENG in direct mode. Similar to the indirect mode, the structure of AB-TENG in the direct mode is designed in a square shape with the same dimensions. The main difference is that the direct AB-TENG has a visible 4 mm × 4 mm square-shaped base–emitter contact point on the surface of the charge-inducing layer. During the alternating motion of the human finger, the change in the electric field causes air breakdown, and the electrons in the human finger flow directly to the collector through the emitter. Even after the skin electrons flow to the collector, the surface charge of the charge-inducing layer will stay negative and attract positive charges in the base terminal due to their physical contact. Therefore, the negative surface charge in the charge-inducing layer repels more electrons from the emitter surface, further enhancing the output. Electrons flowing directly from the base terminal to the collector can significantly reduce energy loss due to the induction process in indirect AB-TENG and increase the energy conversion efficiency of the AB-TENG. Besides, the internal impedance of the AB-TENG in direct mode is less due to the direct flow of electrons. A detailed schematic of AB-TENG in direct mode, illustrating potential distribution and electron flow during the alternating motion of the human finger, is shown in Fig. S6. The electrical equivalent circuit of AB-TENG in direct mode is shown in Fig. 2c.

The electrical performance of AB-TENG in direct mode was studied under a maximum contact force of 24 N. The direct AB-TENG produces a maximum voltage of 290 V and a current of 210 µA (Fig. 2d, e). The measured electrical output was irregular due to the uneven contact force from the human finger, leading to inconsistent electron flow. Therefore, the reliability of the device output was verified using four direct AB-TENGs (samples 1, 2, 3, and 4) with similar dimensions and working conditions. Figure S7a shows that there is *a* ± 3% deviation from the peak output of standard AB-TENG, which is quite negligible compared to the electrical performance. Furthermore, the electrical output of an AB-TENG was investigated with four different individuals (A, B, C, and D) under a maximum contact force of 24 N. There exists an approximate voltage and current difference of 15 V and 10 µA, respectively, between the individuals, as shown in Fig. S7b. However, it has less significance on the output. Next, the electrical performance of AB-TENG was optimized by varying the external load resistance. As the resistance increases, the output voltage increases gradually, whereas the current reduces, as shown in Fig. 2f. The AB-TENG produces a peak power of 22 mW at 8 MΩ load resistance (Fig. S8a). In TENG, the contact–separation process generates an empty interval between the peak outputs. These peak outputs have different time durations according to load resistance. When peak time is considered, the average power generated by AB-TENG will vary. Therefore, a transient response analysis of the AB-TENG output is conducted using the voltage spike, as shown in Fig. 2g. The voltage output exhibits a sharp rise in the peak due to the instantaneous ESD of electrons from the finger and has a long duration under open-circuit conditions. However, the plots in Fig. S9 show that the output voltage time duration varies with load resistance. The AB-TENG produces a sharper voltage spike with a microscale peak period at 50 Ω load resistance. As the load resistance increases from 5 to 500 kΩ, the AB-TENG produces a sharper and higher peak output with a millisecond time duration. In this case, RMS values are used to calculate the power without the influence of the peak period. Therefore, the RMS voltage and current output of AB-TENG are directly measured from the oscilloscope at various load resistances, as shown in Fig. S10a. AB-TENG generates a maximum average power of 3 µW at 10 MΩ load resistance (Fig. S10b). Though the RMS power is quite low, the peak power is primarily accounted for in studying the device performance and powering portable electronics.

Since the charges accumulated on the skin are discharged on repeated finger approaches, the study on the durability and long-term stability of the AB-TENG is crucial for investigating its performance. Therefore, an experiment to examine the stability of charge accumulation over time is conducted by tapping the base terminal continuously for 2000 cycles. Figure 2h shows that the performance of the AB-TENG is stable and durable under long-term operations, with the subsequent cycle to peak output showing a small decay. Moreover, the pre-accumulated charges in the body are not fully discharged in each contact–separation process. Rather, the ESD process primarily depends on the electron affinities of the contact materials. The influence of potential difference due to electron affinities between the finger and base terminal is experimentally investigated through two different case studies. In case 1, the base terminal with a copper electrode is replaced with the aluminum electrode; the output of AB-TENG is slightly less due to the reduction in the potential difference between the finger and the base terminal due to the low electron affinity of aluminum compared to the copper electrode (Fig. S11a). In case 2, when two different individuals (A, B) are connected in series as the charge generators, a larger number of positive charges are conducted by the human body to the finger surface of the person (A) who is in contact with the base terminal (Fig. S11b). Therefore, by combining the static charges of two different individuals (A + B), AB-TENG produces a high output due to a larger potential difference (Fig. S11c). Based on experimental investigations, it is clear that the direction of electron transfer in the human body is primarily determined by the relative electron affinities of the finger (low) and the base electrode (high), and the electric potential gradient between them at the moment of contact. Furthermore, the electric field in the charge-inducing layer influences the surface charge and creates potential differences between the emitter and the collector. To experimentally investigate its influence, the charge-inducing layer was tested with different materials (Fig. S8b). The AB-TENG produces different voltage peaks depending on the surface charge of the charge-inducing layer. Negative triboelectric materials, namely PTFE and Kapton, produce a higher positive voltage peak due to their negative surface charge (Fig. S12a). However, the peak voltage of Kapton is less than that of PTFE due to its low surface charge, which results in reduced charge flow. In contrast, when the positive triboelectric materials (polyamide and paper) are used as charge-inducing layers, the AB-TENG produces a higher negative voltage peak due to their positive surface charge (Fig. S12b). Since the polyamide surface charge is high, the AB-TENG with polyamide as a charge-inducing layer produces a higher negative peak than paper. Based on experimental investigations, the electron flow can occur in both directions depending on the positive/negative charge of the charge-inducing layer.

### Performance Analysis of AB-TENG Due to Air-Breakdown Effect

**Fig. 3:**
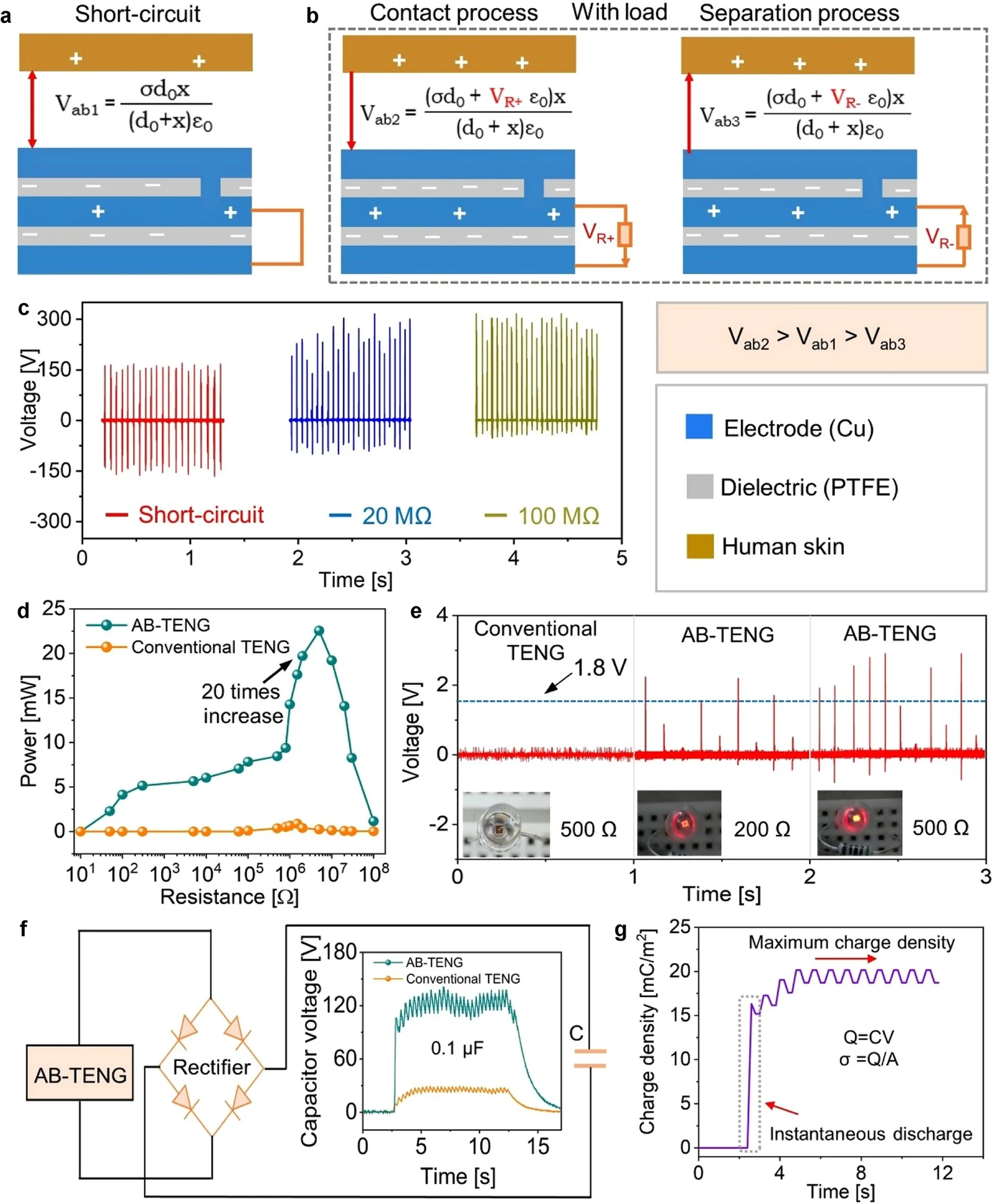
Performance analysis of AB-TENG under short-circuit and load conditions. **a, b** Schematic diagram showing the influence of load on the air-breakdown voltage (V_ab_) in AB-TENG. **c** Output voltage produced by AB-TENG without and with resistive loads, 20 and 100 MΩ. Comparison of AB-TENG with conventional TENG with load test. **d** Comparison of the peak power produced by AB-TENG with the conventional TENG. **e** Voltage output of conventional and AB-TENG at 200 and 500 Ω (low) load resistance. **f** Performance study by charging a 0.1-µF capacitor using AB-TENG and the conventional TENG. **g** Maximum charge density of the AB-TENG

The dynamic electron transfer process in AB-TENG is comprehensively analyzed during the contact and separation of the human finger. The basic working mechanism is the ESD of electrons from the human skin to the base terminal of AB-TENG. Therefore, there is a high breakdown voltage (*V*_ab_) in air due to an intense electric field in the air gap between the finger and the base terminal during the contact process. This output characteristic is studied by measuring the *V*_ab_ with external load resistances. Due to the presence of an external load, the dynamic electron transfer in the external circuit depends on the resistance value and thus differs significantly during both the contact and separation processes [62]. To investigate the dynamic electron transfer process, the *V*_ab_ across AB-TENG is measured under short-circuit and load conditions (Fig. 3a-c). The short-circuit condition results indicate that *V*_ab1_ during the contact process produces a positive value that is equal to the negative peak produced during the separation process. However, the output characteristics of AB-TENG under load conditions differ significantly. The dynamic electron transfer process is analyzed with external loads of 20 and 100 MΩ as examples. The results indicate that the instantaneous electron transfer from the emitter to the collector during the contact process will result in an additional positive voltage (VR +) across the load, causing *V*_ab2_ to be higher than *V*_ab1_. However, the electron transfer from collector to emitter during the separation process will result in an additional negative voltage (VR-) across the load, causing *V*_ab3_ to be smaller than *V*_ab1_. Therefore, the air-breakdown voltage is higher during the contact process. It is noted that an increase in load resistance produces an increased *V*_ab_ during contact, but a reduced *V*_ab_ during separation. Thus, the increased voltage across the air gap will accumulate and enhance the ESD process.

To demonstrate the advantage of the air-breakdown technique, the AB-TENG is compared with the conventional tactile TENG, fabricated in the single-electrode mode (Fig. S13a). In the conventional TENG, the dielectric layer is placed at the top of the device and is exposed to the atmosphere. During the contact and separation process, the human skin forms an electric field between the skin and the dielectric layer (Movie S1). To convert this electric field into electricity, an electrode is fixed below the dielectric layer. When human skin contacts the dielectric layer, the tactile TENG produces electrical output through contact electrification and the electrostatic induction process. However, due to the indirect electrostatic induction process, the electrons from human skin are wasted in the air and are not effectively converted into electricity. Hence, the power conversion efficiency of the conventional tactile TENG is minimal. Unlike conventional TENGs, the AB-TENG structure is designed by placing an electrode (base terminal) at the top of the dielectric layer (charge-inducing layer) to collect human skin electrons through the air-breakdown effect. The electrical output produced by AB-TENG, during the contact and separation of a human finger, is compared with the conventional tactile TENG (Fig. S13b). The output voltage produced by AB-TENG in direct mode is around six times higher than that of conventional tactile TENG. More notably, the output voltage of AB-TENG in indirect mode is almost three orders of magnitude larger than that of the conventional TENG. In general, conventional TENGs have high impedance and produce an extremely low voltage with low load resistance. On the other hand, the AB-TENG has a low internal impedance due to the direct discharge of electrons from the finger, generating significant voltage even under low load resistance. To experimentally verify the influence of load on the electrical output, an impedance test was performed between the conventional TENG and the AB-TENG. As the load resistance increases from 10 Ω to 100 KΩ, the conventional TENG produces a significantly lower voltage, while the current reduces gradually (Fig. S14a). Therefore, the output power of conventional TENGs is not suitable for powering electronics requiring a threshold voltage at low resistance (Fig. S14b). On the other hand, the voltage output of AB-TENG gradually increases from 10 Ω to 100 KΩ, whereas the current reduces gradually (Fig. S14c). A quantitative comparison of output power produced by AB-TENG and the conventional TENG is conducted under matched impedance conditions (Fig. 3d). The maximum power of AB-TENG in direct mode is almost twenty times higher than that of conventional TENG. While the AB-TENG produces a peak power at the matching impedance, it can generate a significant output power even at low resistance, suitable for low-power electronics, as shown in Fig. S14d. The power generation of AB-TENG at a low impedance is verified by powering a red LED, which actually requires a forward voltage of 1.6–2.2 V to operate. The TENGs are connected to power a red LED through parallel 200 and 500 Ω resistances, as shown in Fig. S15. The output voltage of the conventional TENG measured across a 500 Ω resistance is less than the forward voltage of the LED; therefore, it cannot power the LED. On the other hand, the voltage output produced by AB-TENG is high enough to light up the LED even at a low resistance of 200 and 500 Ω, as shown in Fig. 3e and Movie S2.

To confirm the ESD, the electrical performance is further compared by charging a 0.1 µF capacitor using the rectified output from the energy harvesters (Fig. 3f). The voltage charging curve of the capacitor (inset in Fig. 3f) demonstrates that the capacitor is instantaneously charged by AB-TENG up to 102 V due to the ESD of skin electrons. Therefore, the AB-TENG exhibits a higher charging rate during the initial contact process than the conventional TENG. Moreover, the continuous contact and separation process charges the capacitor up to 130 V, which is very high compared to that of conventional TENG (8 V). Based on the capacitor voltage charged by AB-TENG, the total charge transferred by AB-TENG is theoretically calculated using *Q* = *C* × *V*. The plot in Fig. 3g shows that the AB-TENG has a maximum charge density of 20 mC m^−2^, while the instantaneous charge density is 15 mC m^−2^, owing to the sudden discharge of skin electrons. The investigations mentioned above, conducted in comparison with conventional TENGs, validate that the ESD of skin electrons through the air-breakdown strategy proposed in AB-TENG. Additionally, AB-TENG in indirect mode also exhibits high performance compared to conventional TENGs due to the enhanced electric field between the charge-inducing layer and the base (Fig. S16a). The performance of AB-TENG is compared with the reported tactile energy harvesters (Fig. S16b). Reported tactile TENGs produced power densities in the range of 0.13, 0.16, 0.5, 2.2, 2.7, and 3.3 W m^−2^, respectively. Considering the air-breakdown technique as a key factor, the AB-TENG effectively converts the human skin’s electric field into electricity, thereby increasing the energy conversion efficiency of the device. The AB-TENG reported in direct mode exhibits a maximum power density of 31 W m^−2^, which is high among the reported tactile TENGs. Moreover, the power density of AB-TENG in indirect mode is high compared to most reported tactile TENGs.

Finally, a comparison table on the state of the art in air-breakdown TENGs is shown in Table S1. It shows that breakdown TENGs often achieve higher charge densities compared to conventional alternating current TENGs, which are limited by the air-breakdown effect itself, while most of the reported air-breakdown TENGs, referred to as direct current (DC) TENGs, operate by utilizing controlled electrostatic breakdown to produce a unidirectional charge flow, in contrast to conventional alternating current (AC) TENGs. On comparing to the reported breakdown TENGs, the output of AB-TENG is equally high except for a few TENGs which produce current in milliamperes. Unlike typical DC-TENGs or breakdown studies, where current generation is mainly based on contact electrification between triboelectric materials [63], the AB-TENG uses electrostatic discharge (ESD) of human skin electrons as a primary source. Therefore, the AB-TENG is highly effective as it achieved the reported maximum output power under 24 N contact force. Moreover, the simple structure and significantly high output make the AB-TENG a perfect choice for building low-force human–machine interface systems, over the reported breakdown TENGs. Thus, the air-breakdown strategy proposed in AB-TENG is utilized as a positive effect to enhance the energy conversion efficiency of the skin electric field.

### Parameters Influencing the Electrostatic Discharge in AB-TENG

**Fig. 4:**
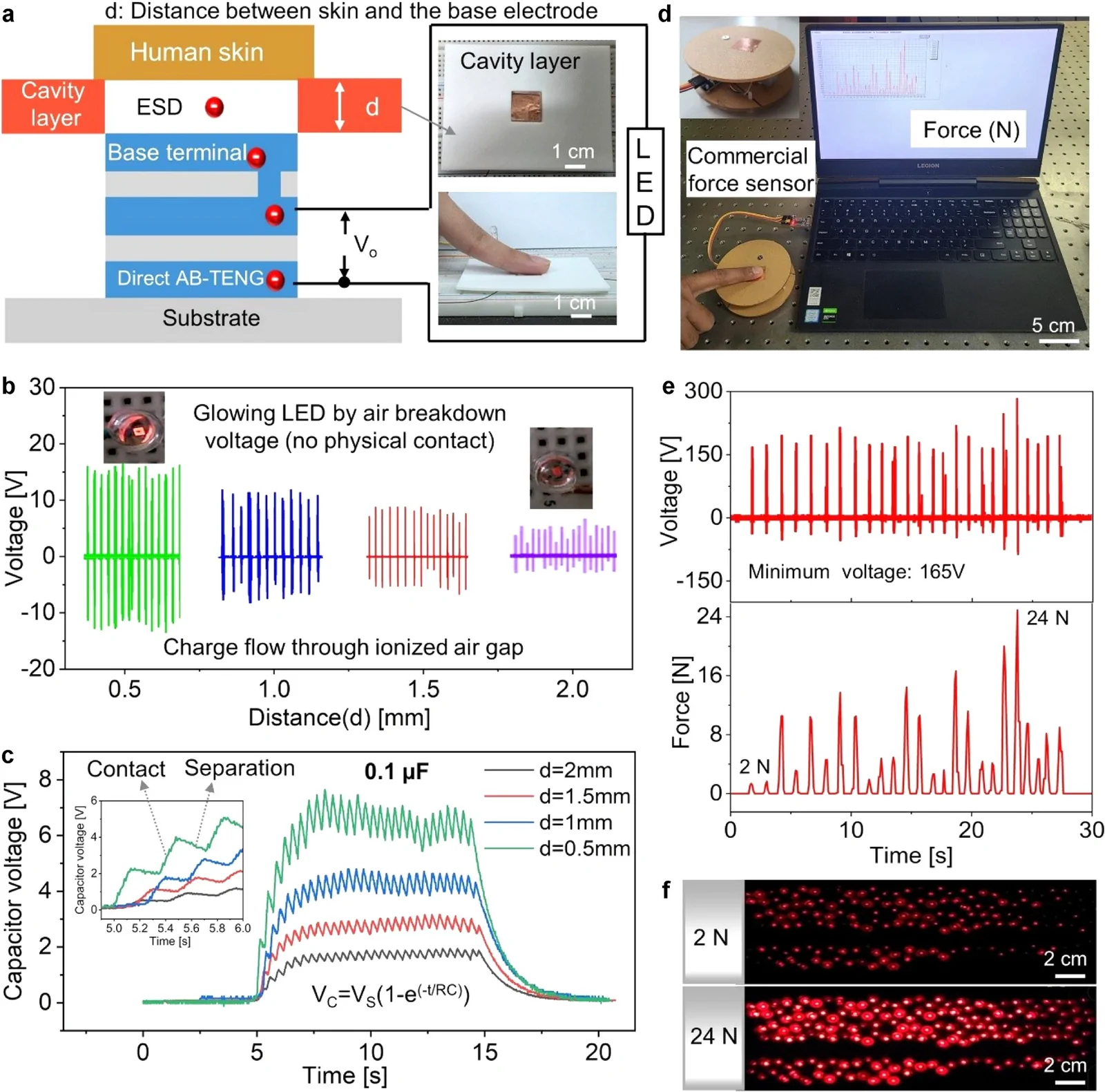
Demonstrations supporting the air breakdown in AB-TENG.** a** Schematic of controlling the distance between human skin and the base terminal. **b** Output voltage with different air gap distances between human skin and the base terminal. (Inset image shows LEDs are powered by the air-breakdown voltage.) **c** Charging a 0.1-µF capacitor with different air gap distances. Impact of contact force between human fingers and the base terminal of the AB-TENG. **d** Image of the contact force measurement system. **e** Relation between contact force and output voltage. **f** Powering 200 LEDs at 2 and 24 N contact force

In an air-breakdown triboelectric nanogenerator (AB-TENG), the ESD of electrons from the human skin to the base terminal takes place through contact and non-contact modes. In non-contact mode, there is no physical contact between the human finger and the base terminal of AB-TENG. Here, the electrons from the finger are transferred through arc discharge due to the air-breakdown effect. As mentioned in the mechanism, according to Paschen’s law, the air-breakdown voltage of direct AB-TENG in non-contact mode depends on distance. Therefore, to validate the air breakdown in non-contact mode, two kinds of experiments were conducted using a substandard and a standard setup. Initially, in the substandard setup, the contact and separation of the human finger toward AB-TENG were tested without the air cavity spacer between the finger and the base terminal. While the distance between the finger and the base terminal could be in a few millimeters, the setup did not have a standard measurement. However, the finger movement toward the base terminal produces an electrical output for directly powering an LED without any physical contact, as shown in Fig. S17a and Movie S3. Next, the air breakdown is tested by measuring the output voltage of AB-TENG, by controlling the distance between the human finger and the base terminal. To standardize the setup, a PLA-based plastic layer was 3D-printed with a square-shaped air cavity having an area of 1.6 cm × 1.6 cm (Fig. 4a). The cavity layer is placed above the direct AB-TENG, having an area of 1.5 cm × 1.5 cm, so that the human finger does not make any physical contact with the base terminal of AB-TENG (Fig. S17b). Besides, the dimension of AB-TENG is less than the air cavity spacer to avoid the influence of triboelectric effects or mechanical deformation of the plastic layer on the underlying device. By varying the thickness of the cavity layer, the distance between the finger and the AB-TENG is controlled (Fig. S17c). The contact and separation of the human finger produce output voltage without any physical contact with the AB-TENG (Fig. 4b). As the distance between the finger and the base terminal is increased from 0.5 to 2 mm, the output voltage of AB-TENG is decreased from 16 to 6 V, owing to a decrease in the strength of the electric field. Though the dimensions of the AB-TENG are reduced from 2.5 cm × 2.5 cm to 1.5 cm × 1.5 cm, the output voltage does not have any significant change. This is because, in contact mode, the electrons are directly discharged to the electrode upon physical contact; therefore, the contact surface area between the finger and the electrode significantly influences the output. In contrast, the electrode surface area has a negligible impact on the output voltage of AB-TENG in non-contact mode, as the electrons are transferred through the ionized air channel. To confirm the electrical output produced by AB-TENG in non-contact mode, a red LED was powered directly with a gap distance of 0.5 and 2 mm (inset in Fig. 4b and Movie S3). The reliability of electron flow through the ionized air gap is further verified by charging a capacitor. Figure 4c shows the voltage charging curve of a 0.1 µF capacitor charged by AB-TENG with different air gap distances between the finger and the base terminal. As expected, the 0.5 mm air gap distance charges the capacitor with a greater voltage than the 2 mm air gap distance. This suggests that a smaller air gap allows electrons to transfer from human skin due to a stronger electric field than a larger air gap distance. The inset in Fig. 4c shows the charging and discharging cycle of the capacitor during the respective down and upward motion of the human finger in non-contact mode.

Next, in contact mode, electrons are transferred from human skin to the base terminal through the contact discharge process. Therefore, the contact force plays a crucial role in enhancing the electrical output of AB-TENG. The contact discharge process in AB-TENG is tested systematically under different contact forces. For demonstration, the direct AB-TENG is fixed on a commercial force sensor to measure the contact force using the computer (Figs. 4d and S17d). The random tapping of a human finger on a force sensor results in a minimum and a maximum contact force of 2 and 24 N, respectively. The relation between the contact force and the corresponding output voltage of AB-TENG is shown in Fig. 4e. At a lower contact force of 2 N, the output voltage of AB-TENG is less due to a less number of electrons transferred to the base terminal, while the larger contact force of 24 N produces the maximum voltage due to the transfer of a large number of electrons to the base terminal. Even though the contact force is very low (2 N), AB-TENG produces a minimum voltage of 165 V due to the strong electric field created by direct contact between the human finger and the base terminal. Furthermore, it is apparent that the AB-TENG could produce a significant electrical output even if the contact force is less than 2 N. The reliability of AB-TENG electrical output at different contact forces is verified by powering 200 LEDs (Fig. 4f), using the circuit connection depicted in Fig. S18. The 2 N contact force lights up the LEDs with lower intensity, while they glow with high intensity at a larger contact force of 24 N (Movie S4).

Apart from the primary parameters (distance and contact force), the effect of environmental factors such as humidity and temperature on the device performance is investigated. Initially, an air humidifier along with a humidity sensor was used to regulate the environmental humidity to a desired value, as shown in Fig. S19a. Under a relative humidity from 20% to 80%, the output voltage of AB-TENG is reduced from 290 to 160 V (Fig. S19b). However, low-humidity levels (< 40%) have a negligible impact on the device output. On the other hand, the humidity levels above 50% significantly degrade the performance of AB-TENG. Since the performance of AB-TENG primarily depends on static skin charges, low-humidity environments are ideal for static charge accumulation because the lack of moisture in the air means there are fewer conductive paths for charges to escape. Contrastingly, a high ambient moisture causes water molecules to adsorb onto surfaces, including skin and AB-TENG materials, forming a thin, conductive water layer. This layer facilitates the rapid dissipation and neutralization of triboelectric charges, which significantly reduces the amount of static energy that can build up in skin. As a result, the electrical output of AB-TENG decreases dramatically in high-humidity environments. Nevertheless, the output of AB-TENG is not entirely degraded even at high-humidity levels. Next, the impact of temperature on AB-TENG output is tested by keeping the device on a temperature-controlled hot plate. The temperature of the hot plate was varied from 20 to 80 °C, and the corresponding voltage was measured, as shown in Fig. S19c. The output shows that the increase in temperature up to 80 °C has less impact on the performance of AB-TENG. Though the high temperature might initially increase contact area and enhance performance, a very high temperature (above 200 °C) could lead to degradation. Besides, the high temperature might influence the AB-TENG performance and skin static energy by altering material properties and skin hydration levels. Moreover, the AB-TENG exhibits high output power at a very low contact force from the finger, ranging from 0 to 24 N. Therefore, the AB-TENG is more suitable for daily finger–machine interface applications, where the average contact force exerted by the human finger ranges from 2 to 15 newtons.

### AB-TENG-based Next-Generation Thin Electronics

**Fig. 5:**
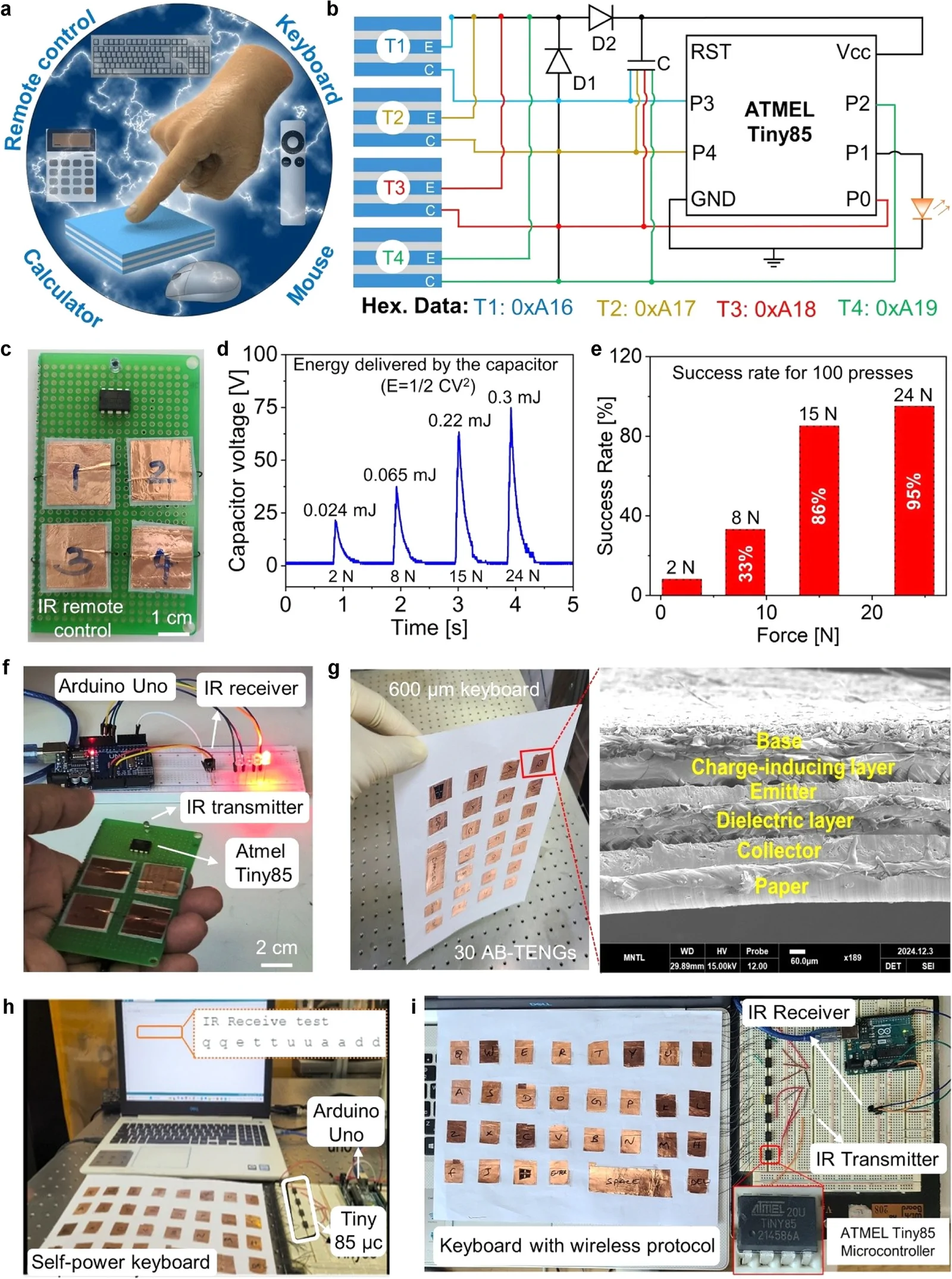
**a** Schematic of AB-TENG for developing next-generation thin electronics. Demonstration of a self-powered IR (infrared) remote controller for controlling four LEDs. **b** Electric circuit for connecting four AB-TENGs to the Atmel Tiny85 microcontroller, and hexadecimal data communication through an IR transmitter. **c** Image of the developed IR remote control. **d** Voltage stored by the capacitor after a single press (with 2, 8, 15, and 24 N), and the corresponding energy delivered by the capacitor. **e** Success rate over 100 presses at 2/8/15/24 N. **f** Images showing the IR remote control of LEDs. **g** Images of the self-powered ultrathin keyboard made on an A4 sheet with a thickness of 600 µm. **h, i** Demonstration of AB-TENG-based next-generation keyboard with wired and wireless (IR) protocol, respectively

The concept of generating electricity from human skin contact represents a promising approach for the construction of next-generation thin electronics based on HMI systems. This method leverages the weak electric field inherent in human skin into electrical energy. Since the AB-TENG can generate electricity from a low contact force of a human finger, it is highly suitable for building day-to-day HMI systems. In particular, the low-power electronics that have frequent contact with human fingers, such as remote controls, keyboards, and calculators, are well suited because their typical contact forces fall within the AB-TENG’s operational range. Integrating AB-TENG with HMI electronics could significantly help to develop next-generation thin electronics (Fig. 5a). Initially, a self-powered infrared (IR) remote control was developed using direct AB-TENG for controlling the LEDs wirelessly (Fig. S20). The transmitter section of the IR remote control was constructed using four AB-TENGs (2 cm × 2 cm), an Atmel Tiny85 microcontroller, a rectifier, a capacitor, and an IR transmitter LED, while the receiver section was built with an IR receiver LED, an Arduino Uno, and four LEDs. The electrical circuit diagram of the transmitter section (Fig. 5b) shows that four AB-TENGs, namely T1, T2, T3, and T4, are electrically connected to four pins of the microcontroller through a rectifier and a capacitor, in such a way that the AB-TENG acts as both a data and a power source. The image of the fabricated self-powered IR remote control is shown in Fig. 5c. Here, the AB-TENGs and IR LED function as remote control buttons and the transmitter, respectively. The electrical signal produced by AB-TENG during the contact of the human finger is not only used as a data signal but also as a power source for the transmitter section. The realistic order-of-magnitude per-press budget to power a short IR transmitter is investigated by measuring the capacitor voltage after a single press and the corresponding energy delivered by the capacitor at a different contact force (2, 8, 15, and 24 N) (Fig. 5d). The capacitor stores 77 V at 24 N on a single press, delivering a maximum energy of 0.3 mJ. On the other hand, the gentle touch of a human finger at 2 N stores 25 V and delivers the lowest energy of 0.024 mJ. Since the power delivered by the capacitor varies with contact force, the power demand by the IR transmitter for the successful operation is tested with its success rate over 100 presses at 2/8/15/24 N, as shown in Fig. 5e. Around 15 N, the success rate is more than 80%, which is nominal for the reliable operation of the IR transmitter. On the other hand, the success rate is less than 33% if the contact force is ≤ 8 N, while it is negligible at 2 N. Moreover, the single press achieves maximum success rate at 24 N, though it is less frequently achieved in day-to-day HMI systems. On successful operation, the microcontroller converts the analog input from AB-TENG into a digital output signal. Further, the analog input from each AB-TENG is differentiated by assigning unique digital codes (e.g., T1:0XA16, T2:0XA17, T3:0XA18, T4:0XA19). Once the human finger contacts AB-TENG, the microcontroller sends the corresponding digital data through the IR transmitter LED. The electrical circuit connection during the operation of individual AB-TENGs is depicted in Fig. S21a-d. Upon receiving digital data from the IR receiver, the Arduino Uno controls the on/off status of the LEDs. The real-time demonstration of AB-TENG as a self-powered remote control for turning on/off the four LEDs is shown in Fig. 5f and Movie S5. Based on our demonstrations, the AB-TENG can be easily used for building a universal IR remote control by changing the unique digital data assigned to each AB-TENG. Therefore, the self-powered IR remote control could act as a universal remote control for TVs, monitors, and music players.

Next, a state-of-the-art next-generation thin electronic device is demonstrated by fabricating the thinnest keyboard based on AB-TENG (Fig. S22a, b). The self-powered keyboard is fabricated on an A4 sheet with 4 rows and 8 columns, having a total of 30 keys. Each key in the keyboard is made from AB-TENG with a thickness of 600 µm (Fig. 5g). When the human finger types the characters, the electrical signal generated from AB-TENG can be used as both a data signal and a power source for the self-powered keyboard. However, the data signal from each character is communicated to the computer in two modes, namely wired and wireless modes. The block diagram for transmitting the data from the self-powered keyboard in the wired mode is shown in Fig. S23a. The self-powered keyboard with the wired connection consists of Atmel Tiny85 microcontrollers, Arduino Uno, and the computer. As a single microcontroller can take only four inputs, a total of 8 microcontrollers, with one assigned to each column, were used for building a self-powered keyboard. During the typing process, the characters typed were sent from AB-TENG to the computer through the microcontroller and the Arduino Uno board (Fig. 5h and Movie S5). Finally, the data typed from the keyboard were sent to the computer using the IR protocol, as shown in Fig. S23b. Using the same circuit components as in the IR remote control (Atmel Tiny85 microcontroller, IR transmitter and receiver, and Arduino Uno), the wireless self-powered keyboard is demonstrated (Fig. 5i). The data signal sent from each key is differentiated by using unique digital data. Based on the assigned digital data, the microcontroller sends the corresponding digital signal through the IR transmitter. Upon receiving digital data from the IR receiver, the Arduino Uno board converts the data into a corresponding character and sends it to the computer monitor. The real-time demonstration of AB-TENG as a self-powered wireless keyboard for sending characters to the computer is shown in Movie S6. In conclusion, we effectively demonstrate that the AB-TENG serves as a promising foundation for developing self-sustaining next-generation electronics applicable in real-world HMI applications.

## Conclusions

In summary, we developed a human skin electric field-induced air-breakdown triboelectric nanogenerator (AB-TENG) with a transistor-inspired architecture. By utilizing electrostatic discharge of electrons from the human finger, the device enables efficient charge collection and conversion through both indirect and direct operating modes. Among them, the direct mode provides outstanding performance, delivering up to 22 mW, 22 times higher than conventional tactile TENGs, and maintaining high output even at low contact forces (165 V at 2 N).

Beyond performance metrics, the AB-TENG demonstrates its practicality through the successful operation of a self-powered IR remote control and an ultrathin 600 µm keyboard capable of both wired and wireless communication. These demonstrations highlight the device’s potential to power real-world human–machine interfaces without external energy sources. Most importantly, this work establishes a new method to utilize skin electrons in tactile nanogenerators by transforming air breakdown, traditionally considered a limitation, into a functional mechanism for efficient energy harvesting. With further optimization, the AB-TENG concept could be extended to large-area, flexible, and wearable electronics, providing a foundation for self-sustaining next-generation HMI and IoT platforms.

## Supplementary Information

Below is the link to the electronic supplementary material.

Supplementary file1 (MP4 3428 KB)

Supplementary file2 (MP4 5169 KB)

Supplementary file3 (MP4 3011 KB)

Supplementary file4 (MP4 2046 KB)

Supplementary file5 (MP4 3270 KB)

Supplementary file6 (MP4 2929 KB)

Supplementary file7 (DOCX 12762 KB)
