# Supplementary material for: Air-Breakdown Triboelectric Nanogenerator Inspired by Transistor Architecture for Low-Force Human–Machine Interfaces
Source: Nanomicro Lett. 2026 Feb 11;18:251. doi: 10.1007/s40820-026-02103-0 (PMC12891284; doi:10.1007/s40820-026-02103-0)
Supplement: Supplementary file 7 — Supplementary file7 (DOCX 12762 KB) [file 40820_2026_2103_MOESM7_ESM.docx]

Supporting Information for

**Air-Breakdown Triboelectric Nanogenerator Inspired by Transistor Architecture for Low-Force Human-Machine Interfaces**

Karthikeyan Munirathinam^1^, Longlong Li^1^, Arunkumar Shanmugasundaram^1^, Jongsung Park^4^, and Dong-Weon Lee^1, 2, 3^ *

^1^MEMS and Nanotechnology Laboratory, School of Mechanical System Engineering, Chonnam National University, Gwangju, 61186, Republic of Korea

^2^ Advanced Medical Device Research Center for Cardiovascular Disease, Chonnam National University, Gwangju, 61186, Republic of Korea

^3^ Center for Next-generation Research and Development, Chonnam National University, Gwangju, 61186, Republic of Korea

^4^ Department of Precision Mechanical Engineering, Kyungpook National University, Sangju, 37224, Republic of Korea

*Corresponding author. E-mail: [mems@jnu.ac.kr](mailto:mems@jnu.ac.kr) (Dong-Weon Lee)

**Supplementary Figures and Tables**


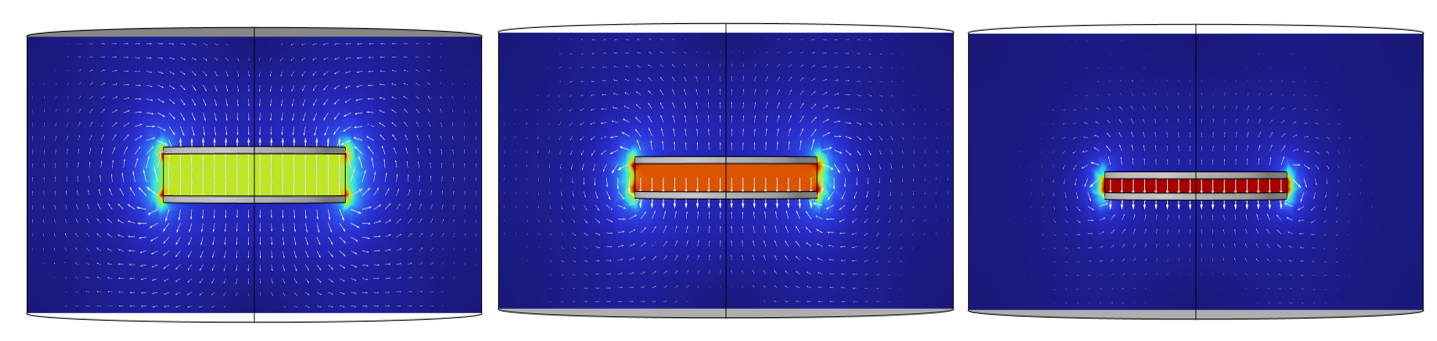


**Fig. S1** COMSOL simulation results for the electric field distribution between the human finger and the base of AB-TENG kept at different distances

**
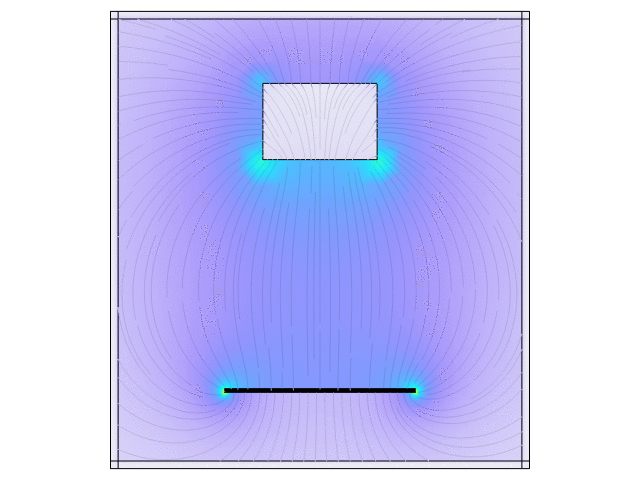

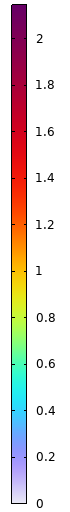
** **(a)**

**(b)**


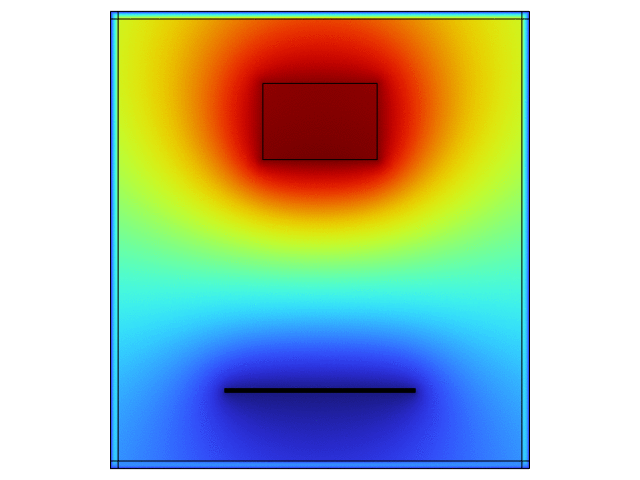

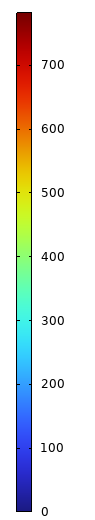


**Fig. S2** COMSOL simulation results (movie) for the (**a**) electric field, and (**b**) potential distribution during the motion of a human finger towards the AB-TENG


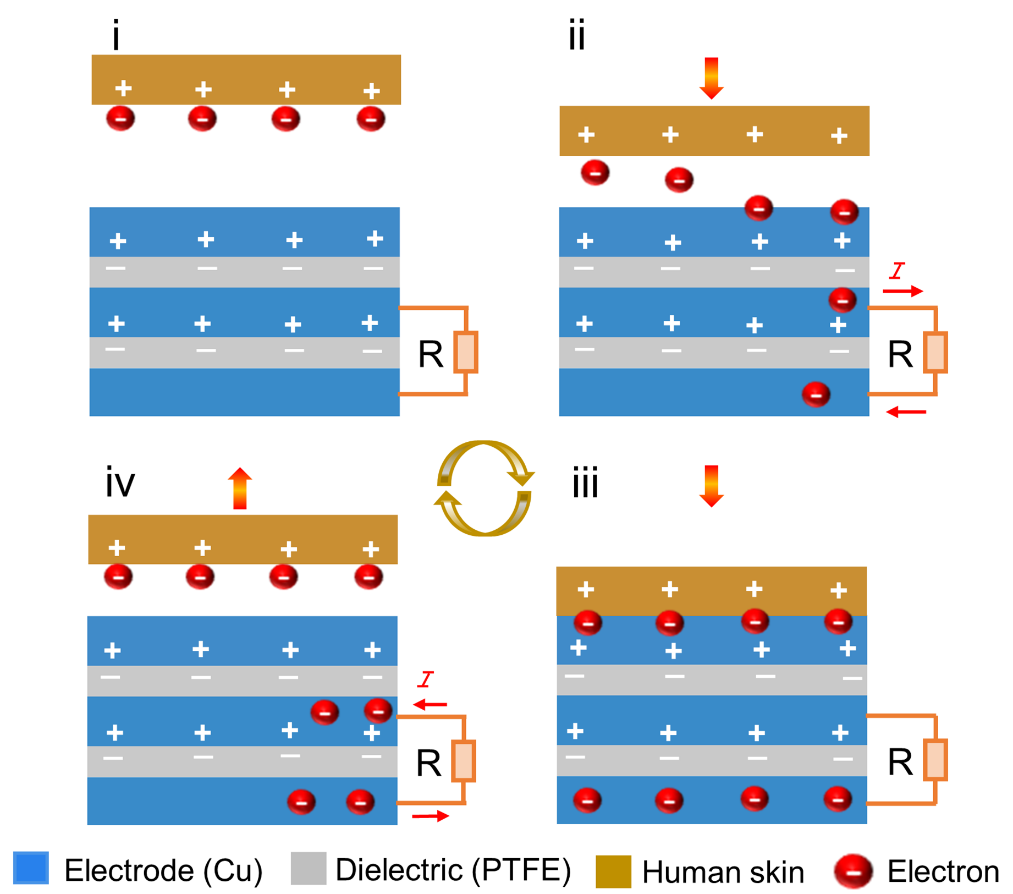


**Fig. S3** Air breakdown induced charge flow mechanism of AB-TENG in indirect mode. (**i-iv**) Alternating charge flow due to the electrostatic induction process between emitter and collector during the contact and separation of the human finger


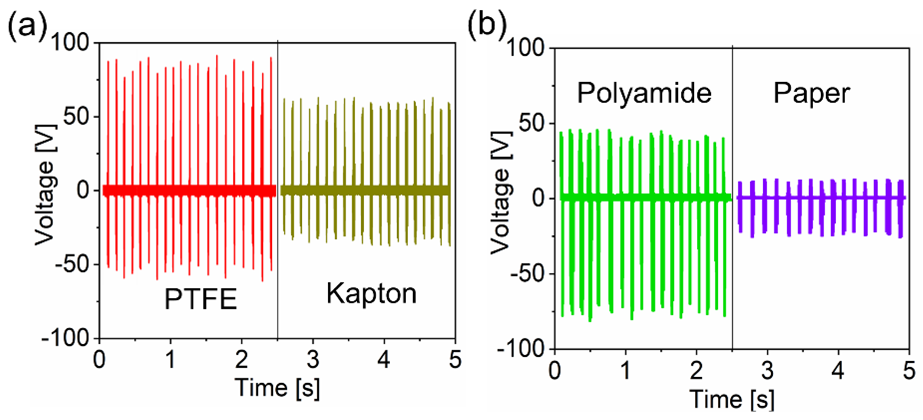


**Fig. S4** Influence of charge inducing layer on the induction process of AB-TENG in indirect mode. Output voltage produced by the AB-TENG by using (**a**) negative and, (**b**) positive triboelectric materials as charge inducing layers


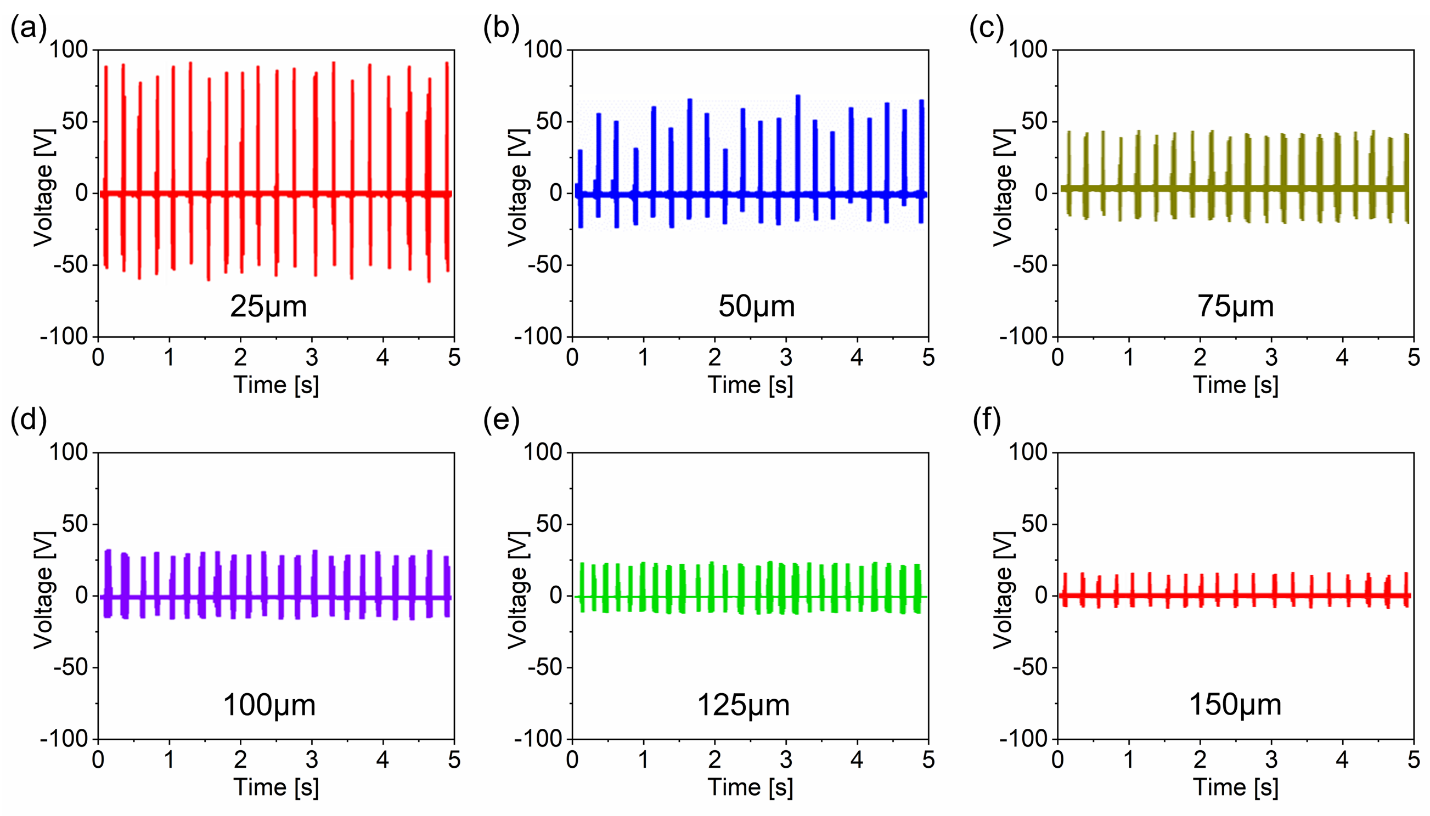


**Fig. S5** Influence of the thickness of the charge-inducing layer on the induction process of AB-TENG in indirect mode. (**a-f**) Output voltage produced by the AB-TENG by using PTFE with different thicknesses from 25-150 µm

c
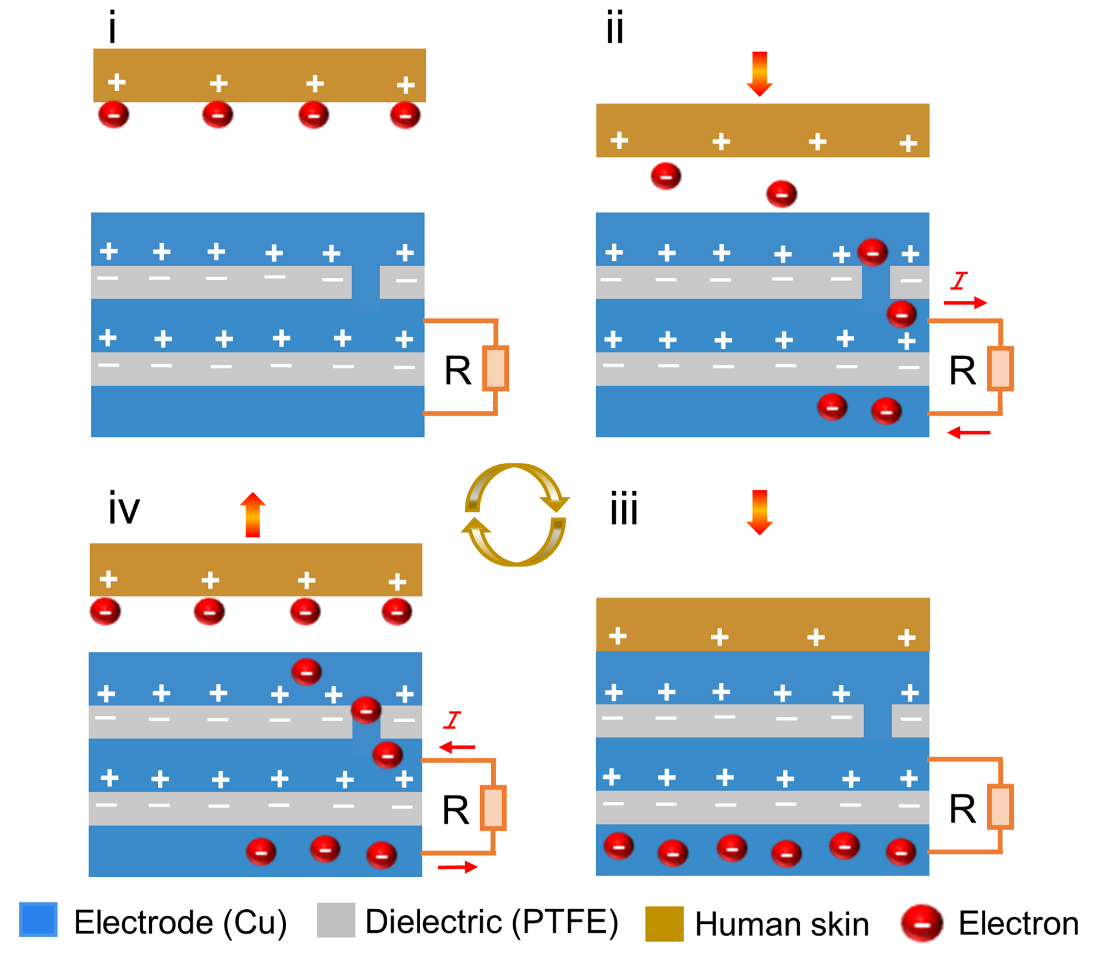


**Fig. S6** Air breakdown induced electrons flow mechanism of AB-TENG in direct mode. (**i-iv**) Alternating electron flow process between emitter and collector during the contact and separation of the human finger


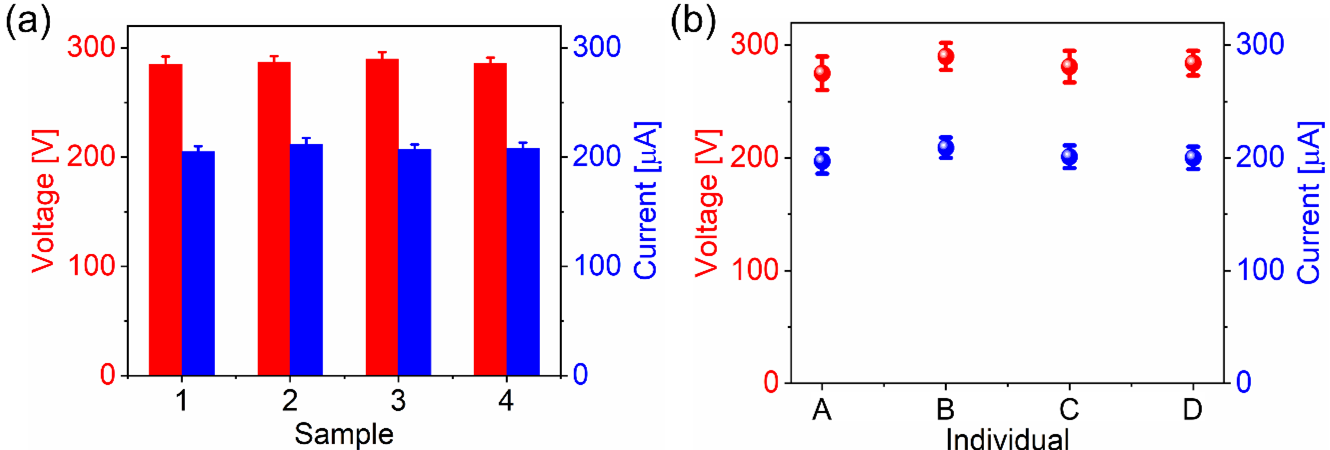


**Fig. S7** (**a**) Electrical output measured by four AB-TENGs (samples 1, 2, 3, and 4) having similar dimensions and working conditions. (**b**) Electrical output produced by AB-TENG with four different individuals (A, B, C, and D) under a maximum contact force of 24 N


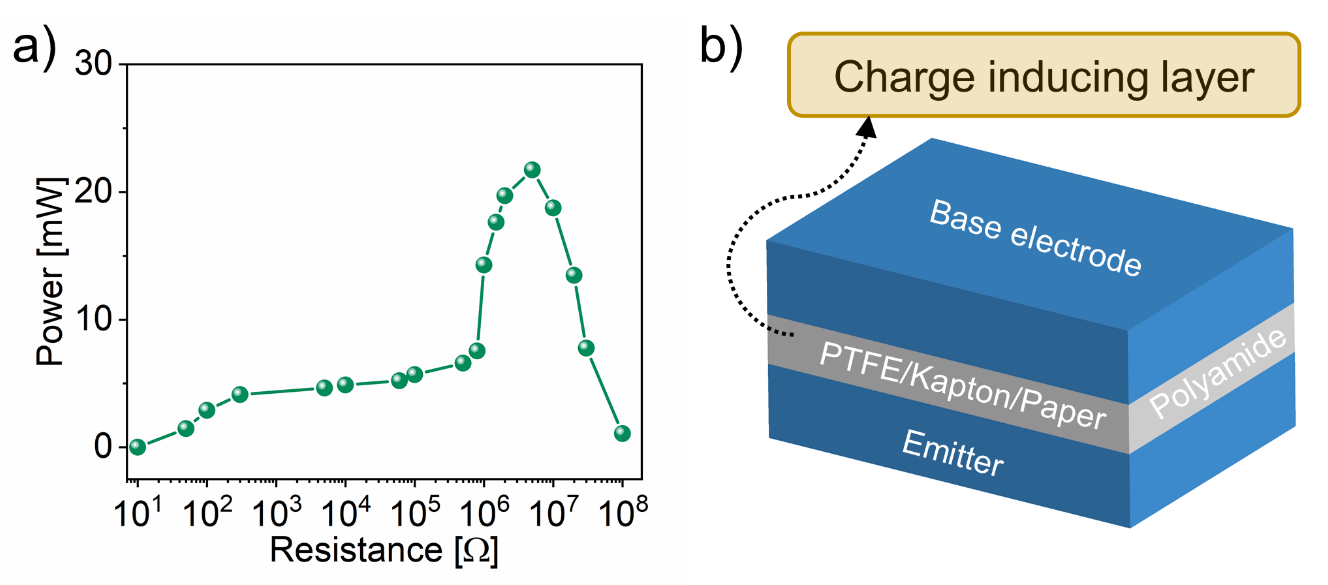


**Fig. S8** (**a**) Peak power produced by AB-TENG at various load resistances. (**b**) Influence of charge inducing layer on AB-TENG performance


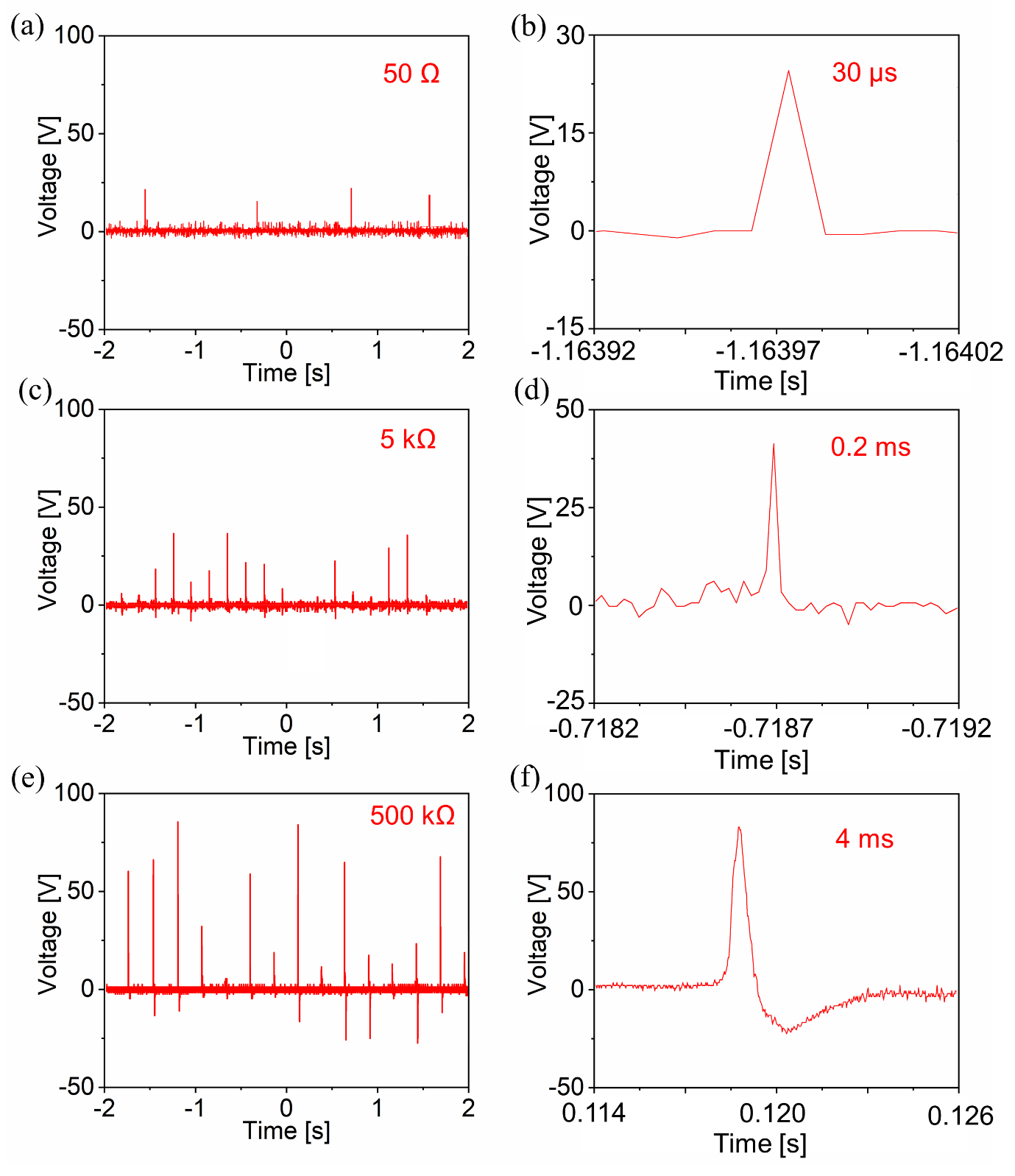


**Fig. S9** Investigation of AB-TENG at a low load resistance. Output voltage produced by AB-TENG at (**a, b**) 50 Ω resistance and peak time duration, **(c, d)** 5 kΩ resistance and peak time duration, **(e, f**) 500 kΩ resistance and peak time duration


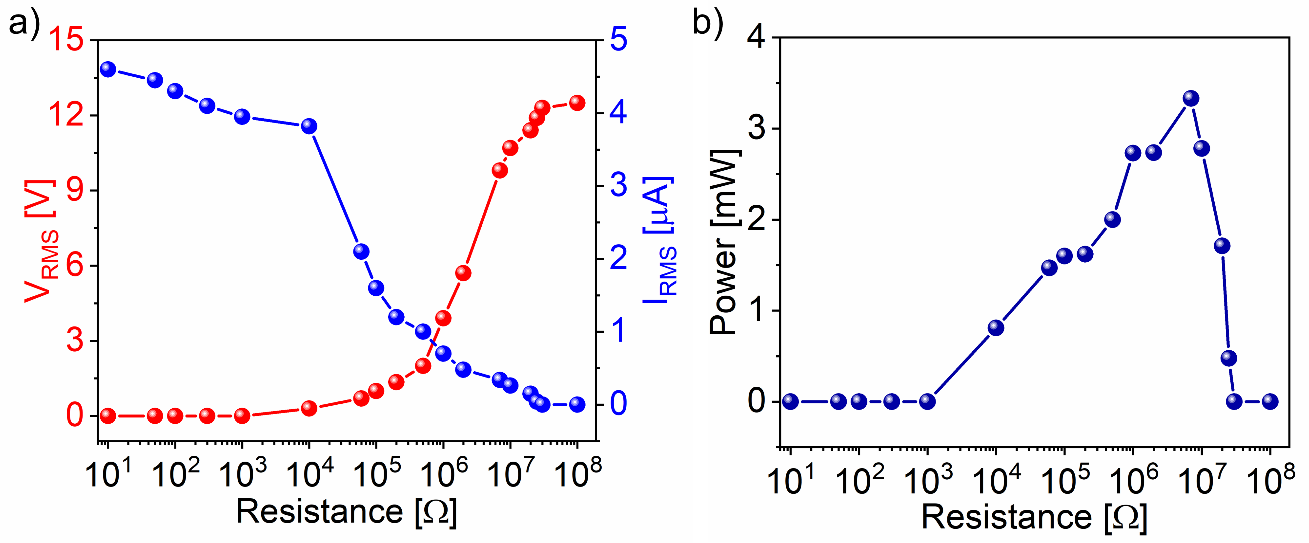


**Fig. S10** (**a**) RMS voltage and current produced by AB-TENG at various load resistances. (**b**) Average power produced by AB-TENG


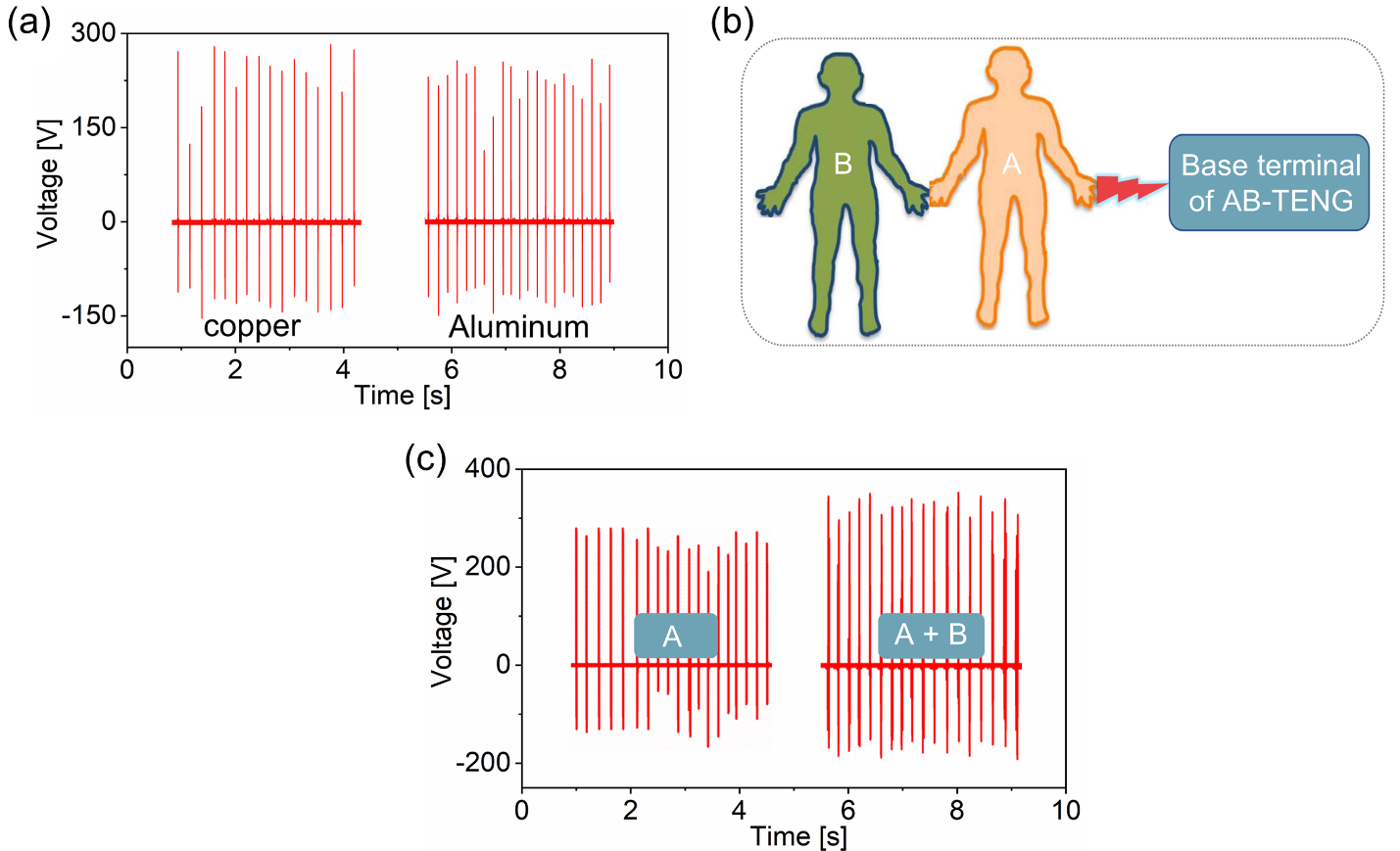


**Fig. S11** Influence of potential difference on the performance of the AB-TENG. (**a**) Influence of materials' electron affinities on the output voltage of AB-TENG with copper and aluminum as a base electrode material. (**b, c**) The impact of two individuals (A, B), connected in series as the charge generators, on the output of AB-TENG


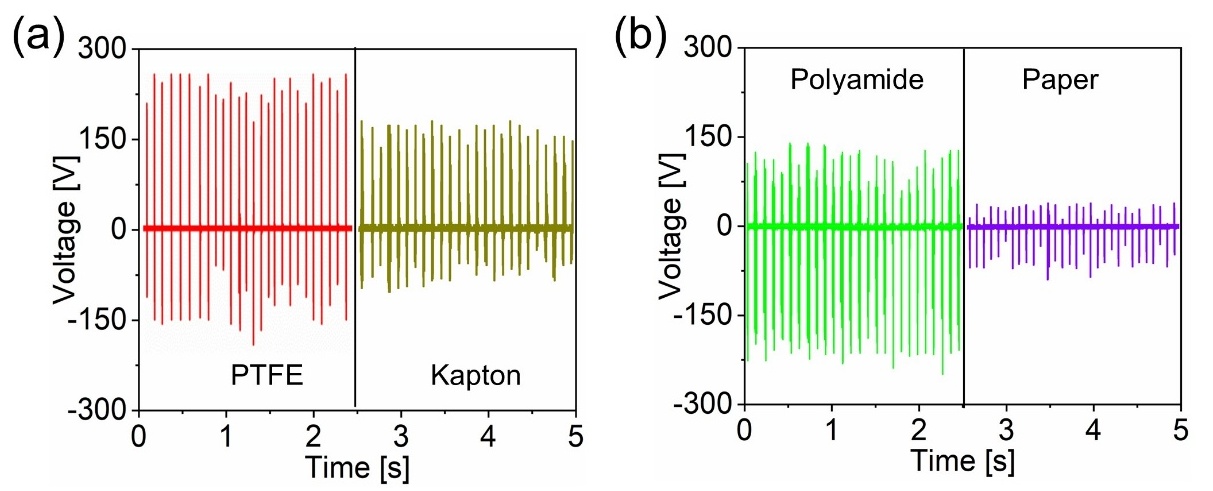


**Fig. S12** Influence of charge-inducing layer on the induction process of AB-TENG in direct mode. Output voltage produced by the AB-TENG by using (**a**) negative and (**b**) positive triboelectric materials as charge-inducing layers


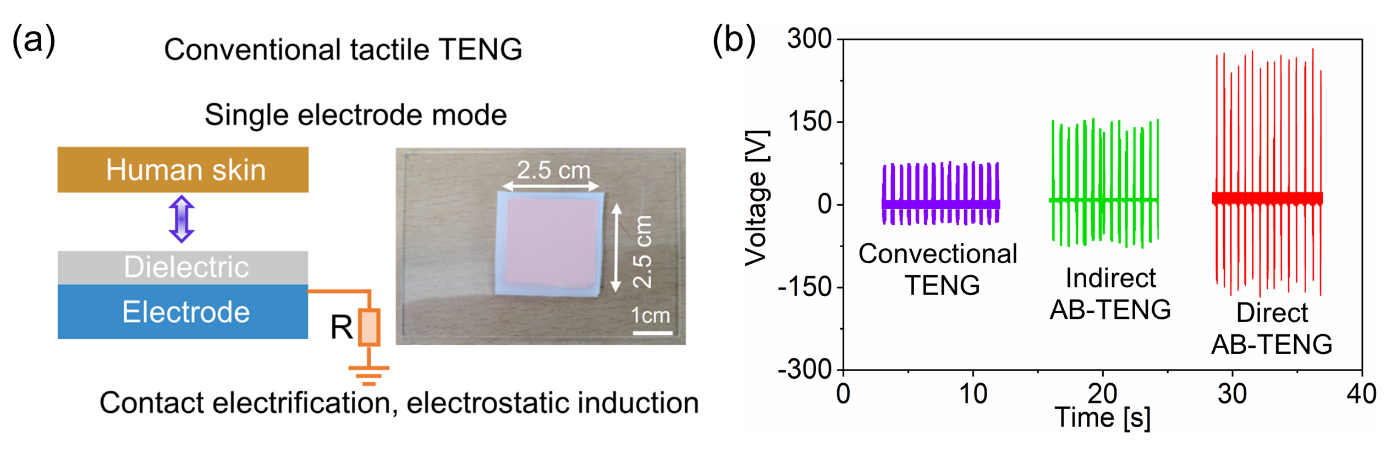


**Fig. S13** (**a**) Schematic and the device image of a conventional tactile TENG fabricated in single electrode mode. (**b**) Comparison of the output voltage produced by AB-TENG and the conventional TENG


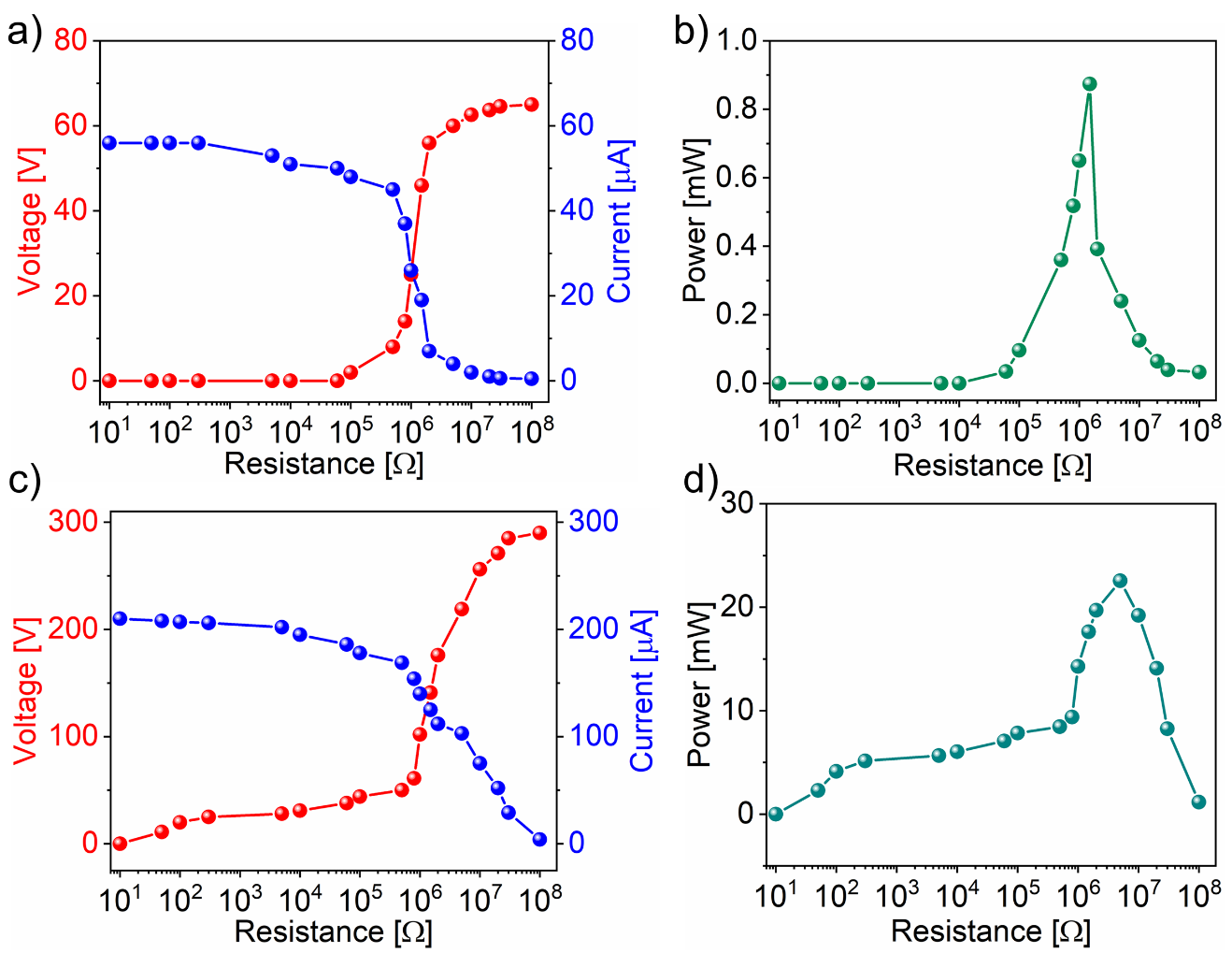


**Fig. S14** Load resistance test (**a**) Conventional TENG output voltage and current trend against the load resistance. (**b**) Peak power produced by conventional TENG. (**c**) AB-TENG output voltage and current trend against the load resistance. (**d**) Peak power produced by AB-TENG


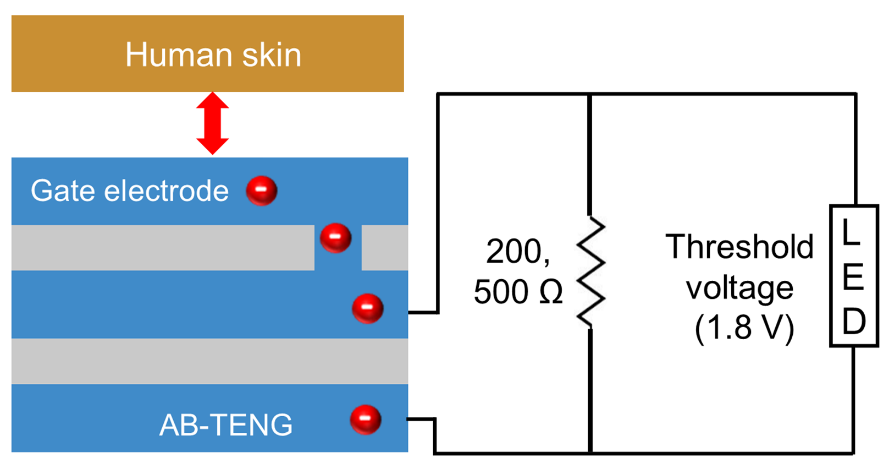


**Fig. S15** Electrical circuit used by conventional TENG and the AB-TENG for powering a red LED (1.8 V threshold voltage) through the parallel connection of 800 Ω load resistance


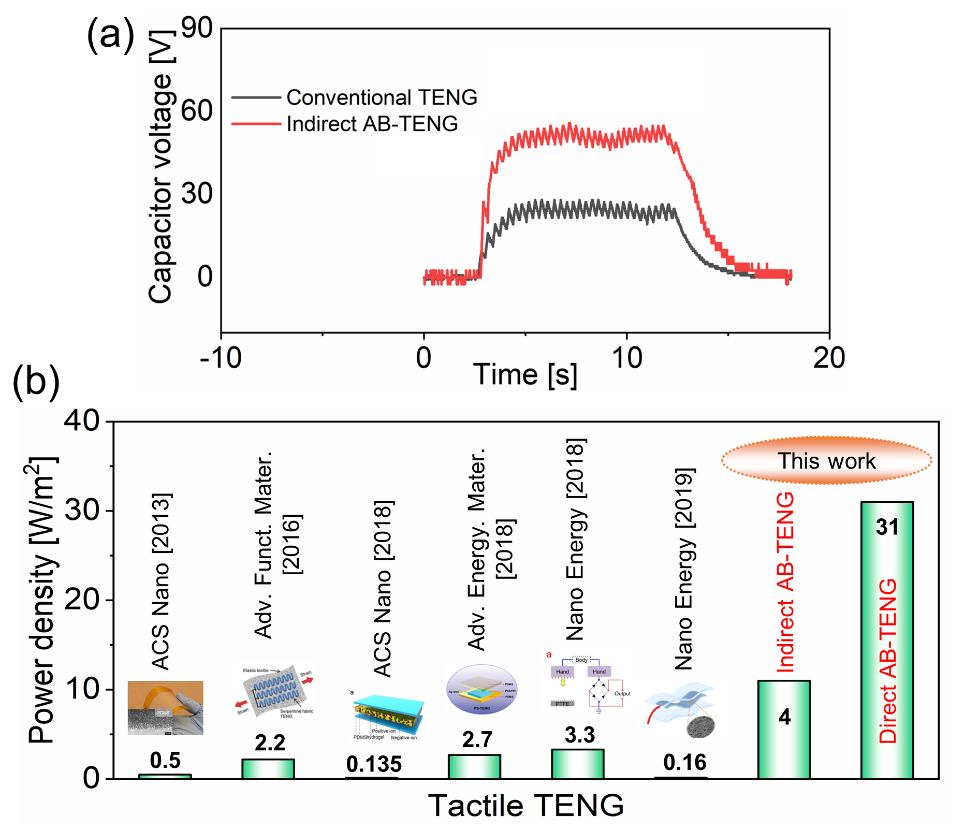


**Fig. S16** (**a**) Comparison of a 0.1µF capacitor voltage charged by using indirect AB-TENG and the conventional TENG. (**b**) Comparison of AB-TENG power density with the reported tactile TENGs

**Table S1** Comparison of AB-TENG with the reported breakdown TENGs

| **S. No** | **Strategy/ Design** | **Mechanism/Key Innovation** | **Voltage (V)/ Current(µA)** | **Power (mW)/Power density (Wm^-2^)** | **Reference** |
| --- | --- | --- | --- | --- | --- |
| 1 | Ion-enhanced Field emission TENG | Field emission due to the triboelectric effect | 500/  250 (mA) | 3.9/NA | Chung et al. [49] |
| 2 | DC-TENG | Triboelectric effect and electrostatic breakdown | 110/15 | 10/NA | Liu et al. [48] |
| 3 | DC-TENG | Triboelectric effect and electrostatic breakdown | 1200/37 | 10/NA | Luo et al. [47] |
| 4 | DLSS-TENG | Triboelectric effect-based controlled field emission | 100/56 | NA/NA | Chung et al. [63] |
| 5 | AB-TENG | Electrostatic discharge of skin electrons | 290/210 | 22/NA | **This work** |


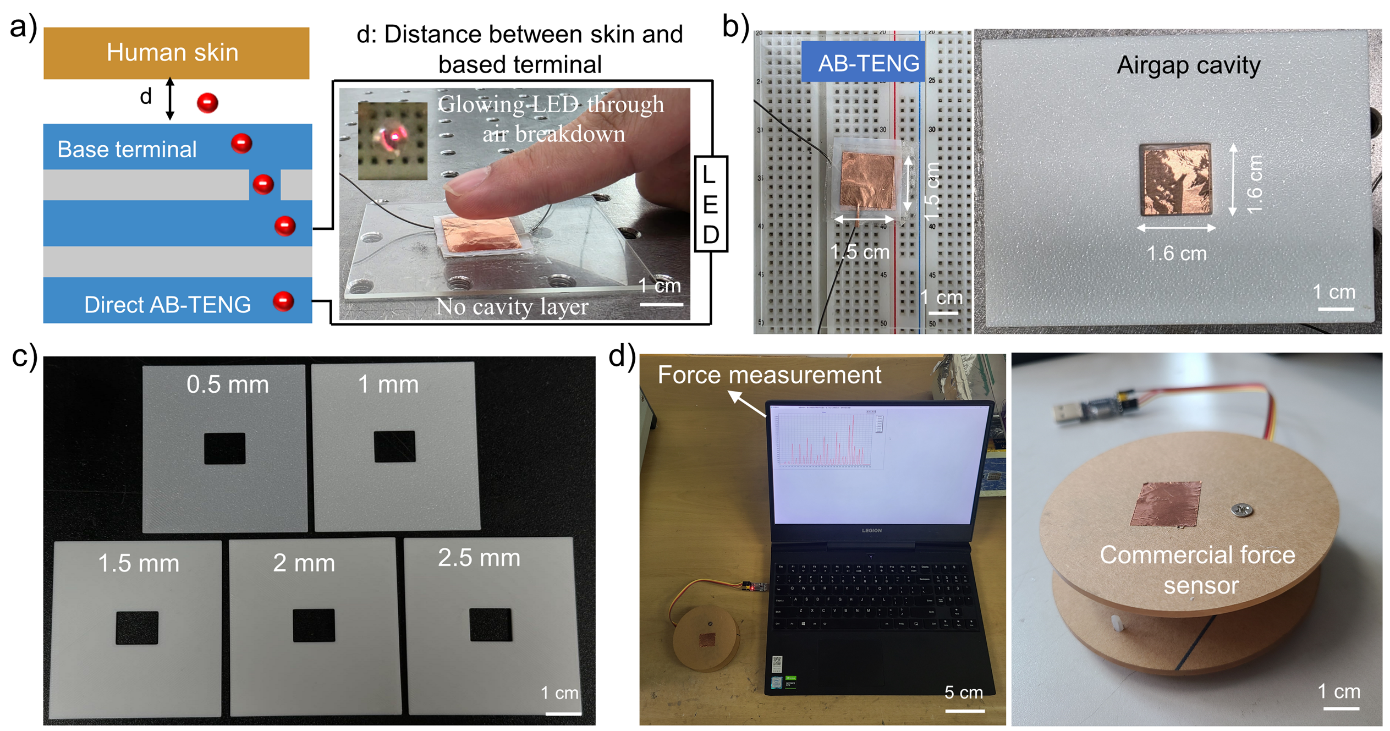


**Fig. S17** Images of the demonstrations conducted to study the influence of airgap distance and contact force on the AB-TENG. **a**) Investigation of air breakdown without using air cavity layers (powering LED). Air breakdown test with a PLA-based air cavity layer. **b**) Image of the fabricated AB-TENG with 1.5 cm x 1.5 cm. Air cavity layer (1.6 cm x 1.6 cm) placed above the AB-TENG. **c**) Air cavity layers made with different thicknesses. **d**) Image of the experimental setup used to measure the contact force


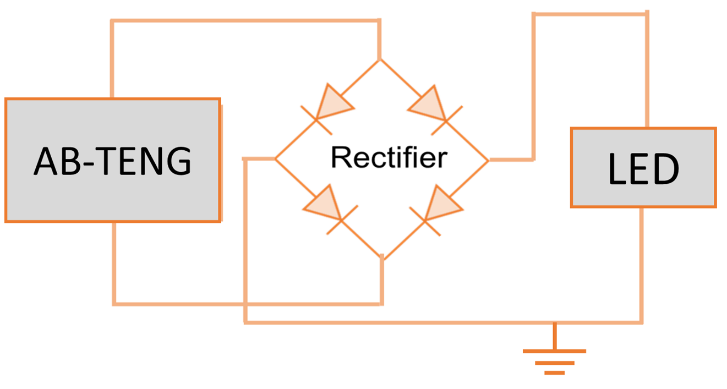


**Fig. S18** Circuit diagram for glowing LED with different contact forces


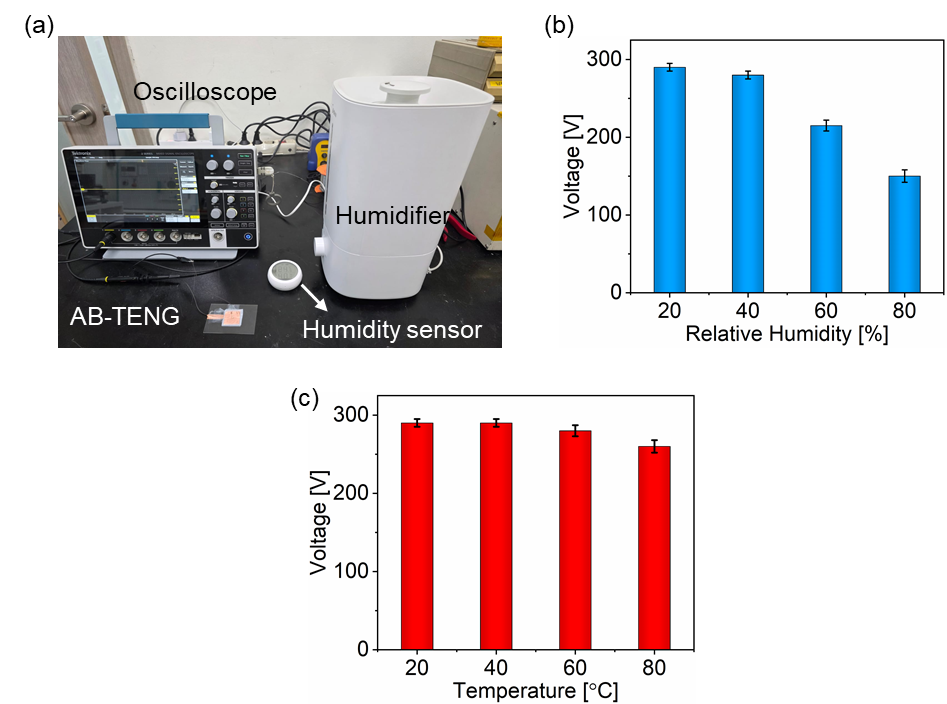


**Fig. S19** (**a**) Humidity experimental setup**.** (**b**) Output voltage variation with respect to relative humidity. (**c**) Performance of AB-TENG under different temperatures


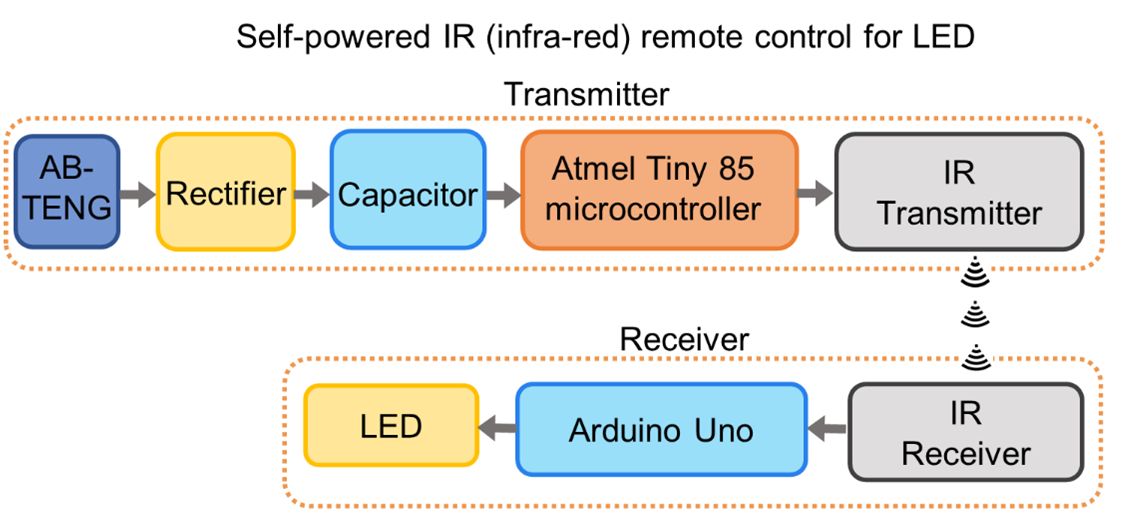


**Fig. S20** Block diagram for the development of an IR remote control using direct AB-TENG


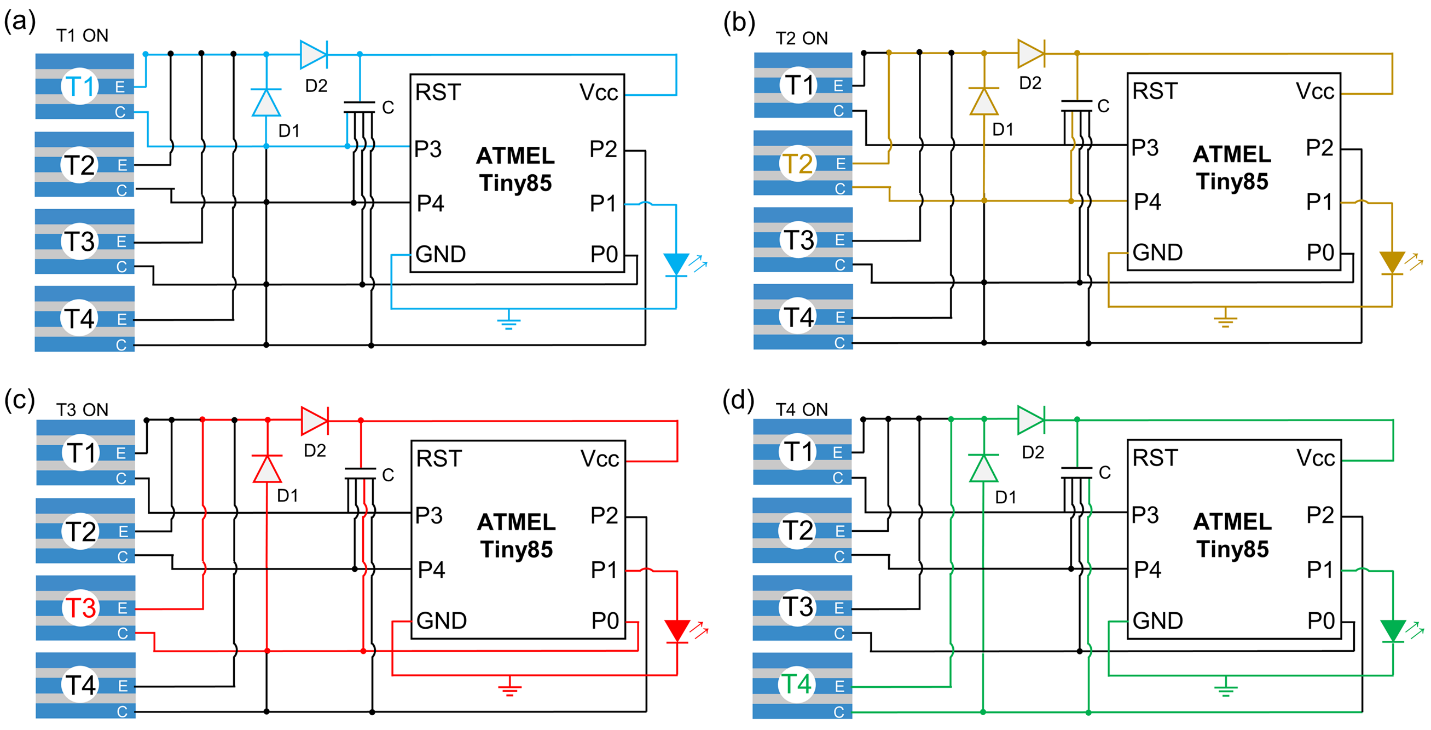


**Fig. S21** (**a-d**) Electrical circuit connection during the operation of individual AB-TENGs (TI, T2, T3, and T4)


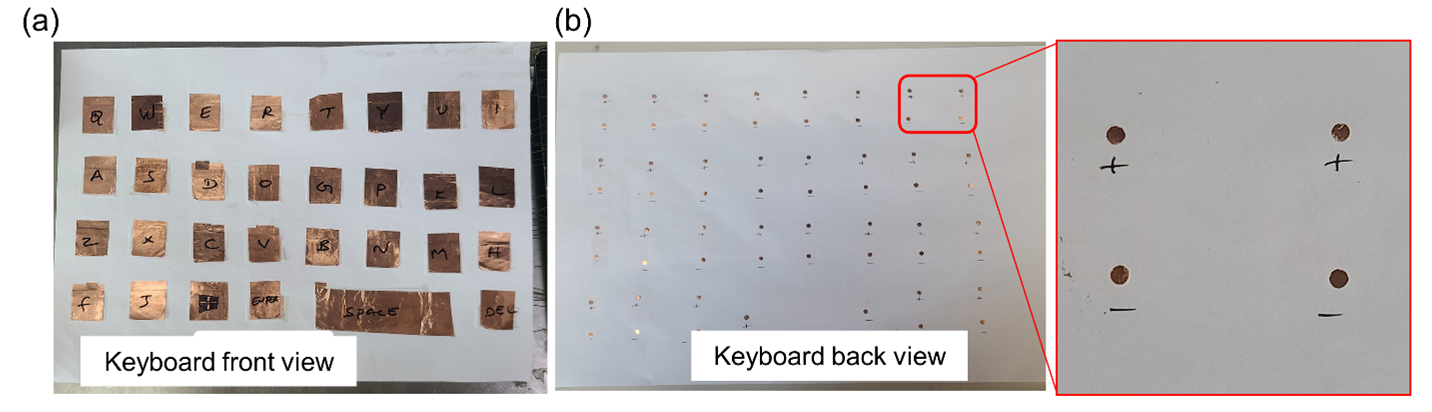


**Fig. S22** Images of the AB-TENG-based self-powered keyboard with 30 characters. **a**) Keyboard front view. **b**) Keyboard back view.


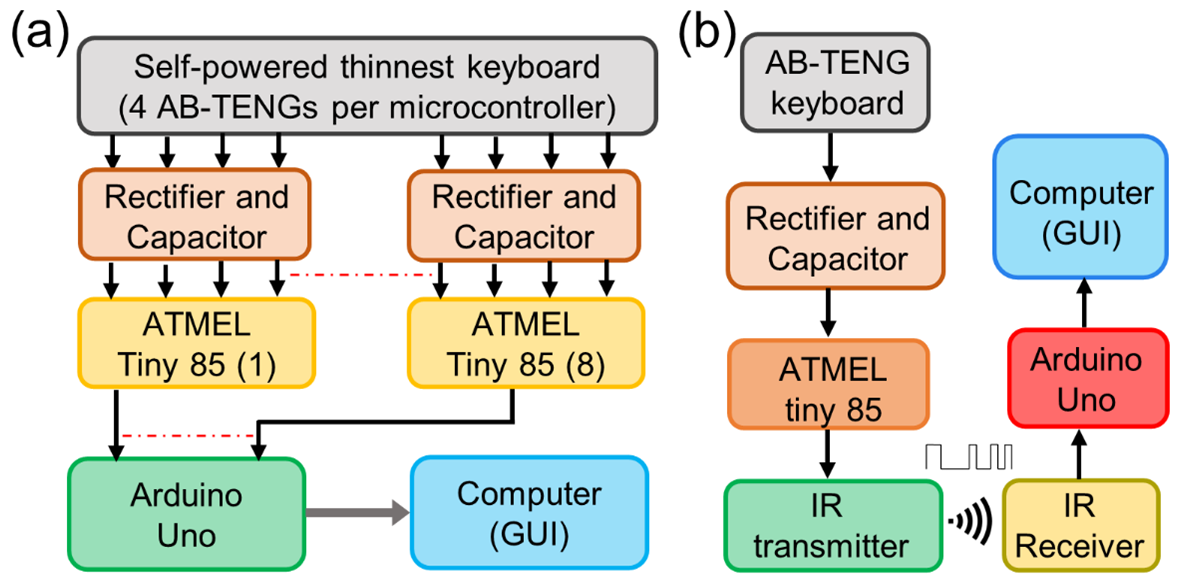


**Fig. S23.** Block diagram for the development of the self-powered keyboard using AB-TENG in (**a**) wired, and (**b**) wireless mode
